# Age, period, and cohort effects on trends in outpatient addiction care utilization in the general Berlin population from 2008 to 2016

**DOI:** 10.1186/s12889-022-12744-6

**Published:** 2022-02-15

**Authors:** Sara Specht, Larissa Schwarzkopf, Barbara Braun-Michl, Nicki-Nils Seitz, Manfred Wildner, Ludwig Kraus

**Affiliations:** 1 IFT Institut Für Therapieforschung, Leopoldstraße 175, 80804 Munich, Germany; 2 Pettenkofer School of Public Health, Ludwig-Maximilians-University, Marchioninistraße 15, 81377 Munich, Germany; 3 Bavarian Health and Food Safety Authority, Veterinärstraße 2, 85764 Oberschleißheim, Germany; 4 Department of Public Health Science, Centre for Social Research on Alcohol and Drugs, Stockholm University, SE-106 91 Stockholm, Sweden; 5 Institute of Psychology, ELTE Eötvös Loránd University, Izabella utca 46, 1064 Budapest, Hungary

**Keywords:** APC analyses, Trends, Alcohol treatment, Drug treatment, Addiction treatment, Substance abuse treatment, Addiction counseling, Substance use disorder, Alcohol use disorder, Illicit substances use disorder

## Abstract

**Background:**

The aim of this study was to decompose independent effects of age, period, and cohort on trends in outpatient addiction care utilization resulting from alcohol (AUD) and illicit substances use disorders (ISUD). Decomposing trends in addiction care utilization into their independent effects by age, period, and cohort may lead to a better understanding of utilization patterns.

**Methods:**

Individuals seeking help in Berlin outpatient addiction care facilities between 2008 and 2016 with an age range of 18–81 years for AUD (*n* = 46,706) and 18–70 years for ISUD (*n* = 51,113) were standardized to the general Berlin population using data from the German Federal Statistical Office. Classification of utilization as AUD- (F10) or ISUD-related (F11, F12, F14, F15, F16, F18, F19) help-seeking was based on primary diagnoses according to the International Statistical Classification of Diseases and Related Health Problems. Age was measured in years and period as year of data collection. Cohort was defined as the mathematical difference between period and age. Age, period, and cohort analyses were conducted using the intrinsic estimator model on AUD- and ISUD-related outpatient addiction care utilization.

**Results:**

Age effects on AUD-related utilization were highest in 18- to 19-year-old and in 39- to 59-year-old individuals. ISUD-related utilization declined almost continuously with increasing age. Period effects on AUD- and ISUD-related utilization were small. AUD-related utilization was highest in cohorts born from 1951 to 1986. ISUD-related utilization increased in cohorts born between 1954 and 1973 where utilization peaked, followed by a decline of the same order.

**Conclusions:**

Age and cohort effects were the strongest drivers of trends in AUD- and ISUD-related outpatient addiction care utilization. Onset of help-seeking in earlier phases of AUD development should be enhanced as well as help-seeking for AUD and ISUD in general. The highest cohort-related rates in the baby boomer and following cohorts for AUD and ISUD underline an increased demand for addiction care.

**Supplementary Information:**

The online version contains supplementary material available at 10.1186/s12889-022-12744-6.

## Background

Addiction care constitutes a relevant sector of health care as alcohol and illicit substance use are important contributors to the global disease burden [1]. As addiction care is a measure to reduce this burden, it is crucial to understand the influencing factors of addiction care utilization and to monitor utilization trends. Knowledge on trends in addiction care utilization may help to tailor and manage demand-oriented care by identifying supply gaps and priority areas.

In Germany, trend analyses of addiction care utilization are scarce. A recently conducted study of the outpatient German Addiction Care Statistical Service unveiled a decreasing proportion of primary alcohol use disorders (AUD) in the last decade. Furthermore, help-seeking decreased for opioid use disorders but increased for cannabis and stimulants use disorders [2]. These aggregated trends are the result of overlapping changes in the age and cohort composition of individuals with substance use disorders and temporal structural changes in addiction care provision that impact on demand for addiction care. A study analyzing cohort effects in a sample of clients seeking help in addiction care services in Berlin showed cohort-related differences in primary substance use disorders [3]. Yet, age and period effects were only accounted for, not estimated.

The composition of the entire population of individuals with substance use disorders is generally not known except for its characteristics based on samples or point estimates of the number of individuals meeting specific criteria such as reporting intravenous substance use or fulfilling diagnostic criteria of addiction. Hence, the evaluation of changes in addiction care management has to start from the general demographic development as the reference population. This reference to the general population is conceptually comparable to the analysis of mortality as the use of addiction care services can theoretically affect anyone. The use of addiction care services reflects the changes in substance abuse in the general population and thus demographic developments against the background of changing substance abuse habits and societal reactions dealing with emerging problems.

Decomposing trends in addiction care utilization into its independent effects by age, period, and cohort (APC) may lead to a better understanding of utilization patterns. Age effects on addiction care utilization reflecting variations in aging processes [4] reveal changes in treatment uptake for specific substance use disorders over the life course. Period effects mirror time-related external influences on all age groups [5] and reveal whether help-seeking behavior changed over time as a result of changes in external conditions such as access requirements or referral pathways. Cohort effects comprise the sum of expositions experienced since the birth of a cohort [6] and demonstrate common experiences of the clientele that might require adjustments in addiction care services. One example is substitution therapy, which has changed from a short-term intervention to a long-term maintenance program (period effect) and created an aging cohort of individuals constantly remaining in long-term opioid substitution treatment (cohort effect). This group did not exist before in addiction care and requires specific therapy aims and case management approaches [7].

In order to investigate temporal changes in addiction care utilization in Germany, the present paper aimed to analyze exploratively the independent APC effects on admission to outpatient addiction care related to AUD and illicit substance use disorders (ISUD) in the general Berlin population.

## Methods

### Setting and procedures

Data came from the outpatient Berlin Addiction Care Statistical Service, a series of annual, cross-sectional surveys of participating outpatient addiction care facilities, predominantly delivering addiction counseling. Facility personnel documented data on their clientele according to a German-wide standardized core dataset [8] containing sociodemographic, disorder- and treatment-related characteristics. We used the surveys from the years 2008–2016. In this period, the same version of the core dataset was applied. The participation rate among registered facilities ranged between 73 and 84% (no participation rates documented before 2012).

Double counting of individuals seeking help more than once per year was accounted for by including only one episode per individual using an identifier variable. Because age was collected at admission, the first rather than the last episode per individual was chosen. In 2010, the identifier variable was missing in 8,388 out of 16,968 cases (including multiple counts) because of a technical malfunction. We treated each of these 8,388 help-seeking episodes as one distinct individual.

### Measures

The primary substance use disorder was defined by codes from the International Statistical Classification of Diseases and Related Health Problems, 10th revision (ICD-10, German modification) using the previous 12 months as the reference time. The number of individuals seeking help as a result of a primary AUD (ICD-10 code F10) served as base for AUD-related outpatient addiction care utilization (AUD-related utilization). Analogously, the number of individuals seeking help for a primary ISUD (ICD-10 codes F11, F12, F14, F15, F16, F18, F19) was used for ISUD-related outpatient addiction care utilization (ISUD-related utilization).

The number of individuals using outpatient addiction care (AUD- or ISUD-related) was standardized to the general Berlin population, generating a utilization rate per 10,000 individuals. The size of the general Berlin population was taken from the German Federal Statistical Office [9]. The general Berlin population was assumed to cover all individuals potentially seeking help in the analyzed facilities because help-seeking in the outpatient setting is regionally restricted to individuals living near the facilities.

Age at admission was measured in years and period as the year of data collection. Cohort was defined as the mathematical difference of period and age.

### Statistical analyses

We portrayed descriptive trends by depicting the number of individuals seeking help for AUD or ISUD in Berlin outpatient addiction care standardized to 10,000 individuals from the general Berlin population.

Estimating APC effects simultaneously is challenging because of their exact linear dependency (period = age + cohort), known as the “identification problem” [10]. To disentangle APC effects on the utilization rates, we used the “intrinsic estimator” approach (for a discussion, see [10, 11]).

For this purpose, the AUD- and ISUD-related utilization rates were each composed in age- and period-specific rates containing age as row, periods as columns, and cohorts as diagonal elements. This resulted in 64 (AUD) and 53 (ISUD) age groups (18; …, 70/81), nine period timepoints (2008, …, 2016), and 72 (AUD) and 61 (ISUD) birth cohorts (1927/1938, …, 1998). Choosing single-year groups instead of summarizing them, e.g., in 5-year intervals, was considered to achieve more reliable results. A wider grouping would have shortened the period range noticeably, resulting in a small number of observations in the analytical models (age*period groups). Although the nine period timepoints limit the extent to alter temporal widths of the APC dimensions within sensitivity analyses, we used 2-year intervals in order to test the robustness of the models (SA1). Help-seeking individuals younger than 18 years (focus on adult population) and older than 70 years for ISUD- and 81 years for AUD-related utilization (too few: less than 10 in summarized age groups) were excluded. The number of individuals seeking help within this age and period range was 46,706 for AUD and 51,113 for ISUD. The general Berlin population totaled 25,297,254 individuals.

The analyzed utilization rates were non-negative count variables with evidence of overdispersion assessed by likelihood ratio tests. Therefore, we applied negative binomial regression instead of Poisson models [12], and the results are presented as incidence rate ratios (*IRR*). The *IRR* and corresponding 95% confidence intervals (*CI*) were plotted along the APC dimensions.

Owing to many empty cells in the APC models and lack of power, distinct illicit substances could not be analyzed separately in the main analysis. To examine substance-specific patterns among the sum of ISUD-related utilization, models with separate help-seeking for primary diagnoses of opioids (OUD, 50.71% of ISUD), cannabis (CaUD, 30.82% of ISUD), and stimulants/cocaine (StiUD, 17.92% of ISUD) use disorders were run as sensitivity analyses (SA2). All analyses were conducted with Stata/SE 15 (Stata Corp LP; College Station, TX, USA) using the “apc_ie” command [13]. An alpha level of 0.05 was used for statistical tests. In addition, the results were also tested using an alpha level of 0.01 (SA3).

## Results

### Descriptive rates

**Fig. 1:**
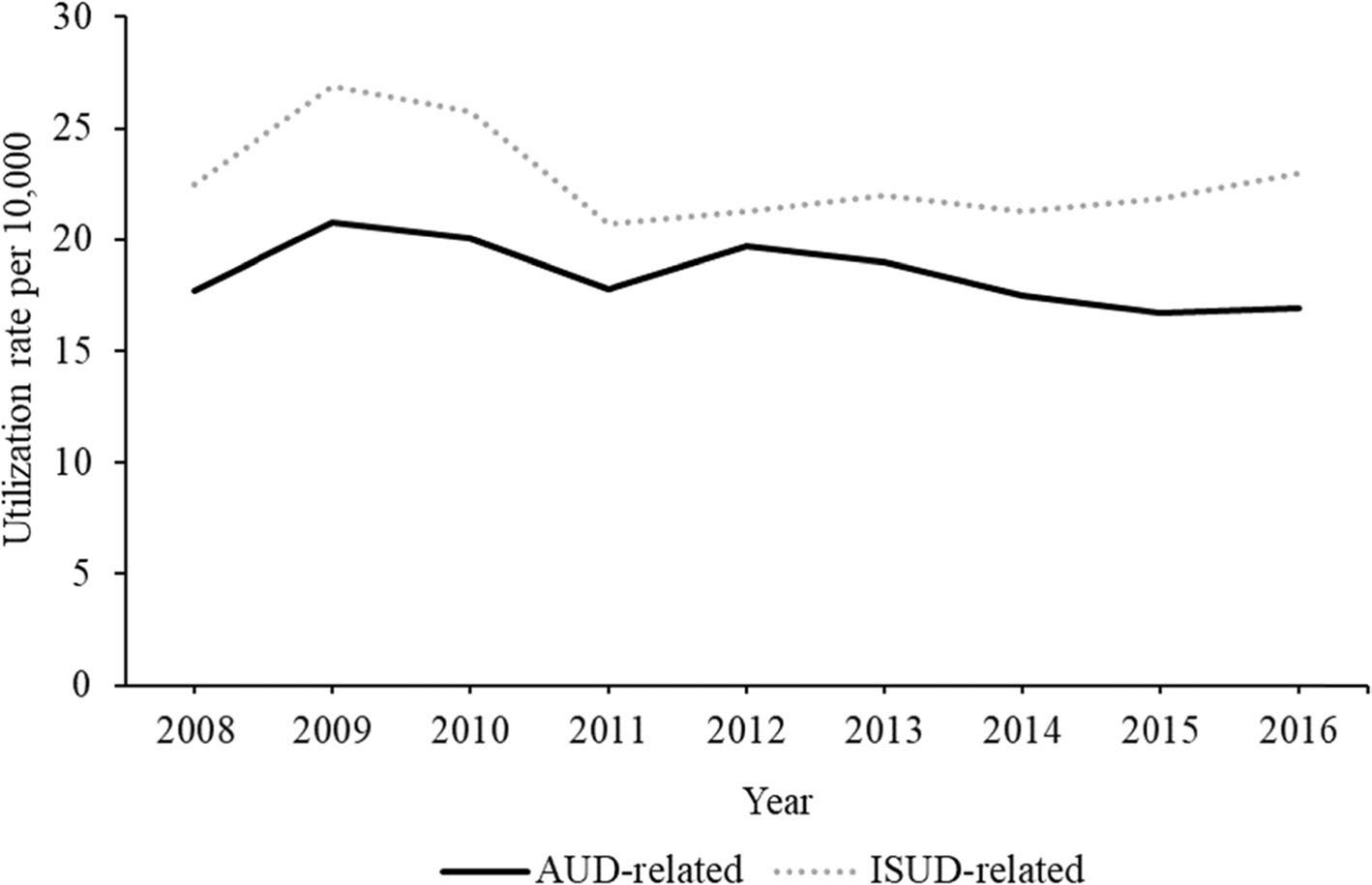
AUD- and ISUD-related utilization rates per 10,000 individuals living in Berlin

Figure 1 presents the AUD- and ISUD-related utilization rates of Berlin outpatient addiction care. Both rates presented an inverse u-shape between 2008 and 2011 with ISUD at an overall higher level. Regarding AUD, there was an increase in 2012 followed by a decline until 2015. From 2012 onwards, ISUD-related utilization rose slowly and almost constantly.

### Intrinsic estimator APC models on AUD- and ISUD-related utilization

#### Age effects

**Fig. 2:**
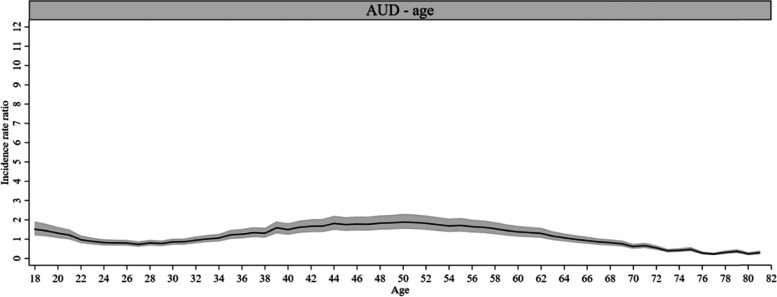
Age effects on AUD-related utilization rate, 1-year age groups (*IRR* and 95% *CI*)

**Fig. 3:**
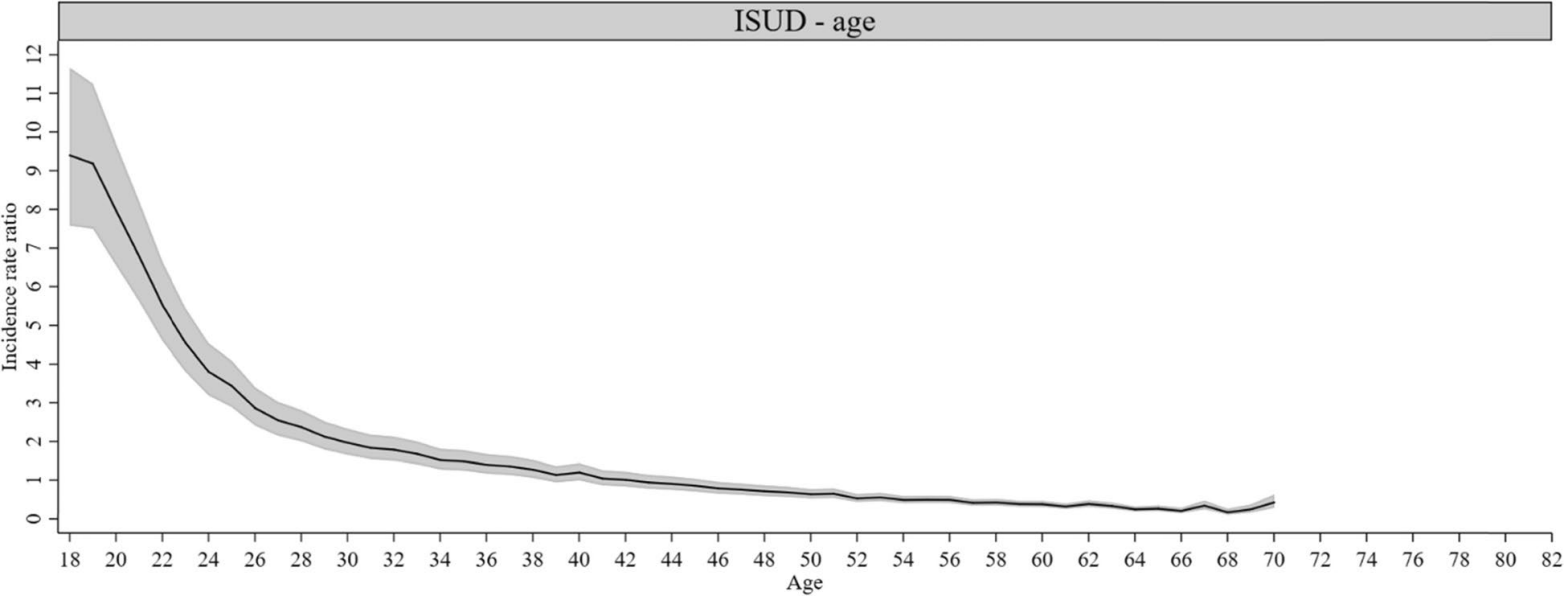
Age effects on ISUD-related utilization rate, 1-year age groups (*IRR* and 95% *CI*)

**Fig. 4:**
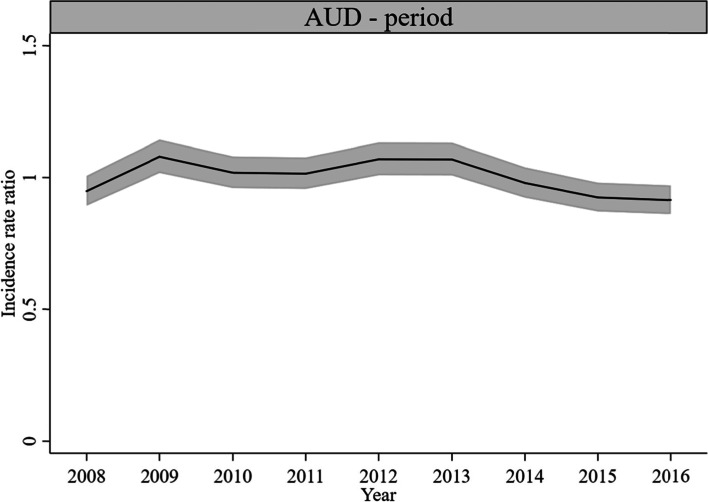
Period effects on AUD-related utilization rate, 1-year period groups (*IRR* and 95% *CI*)

**Fig. 5:**
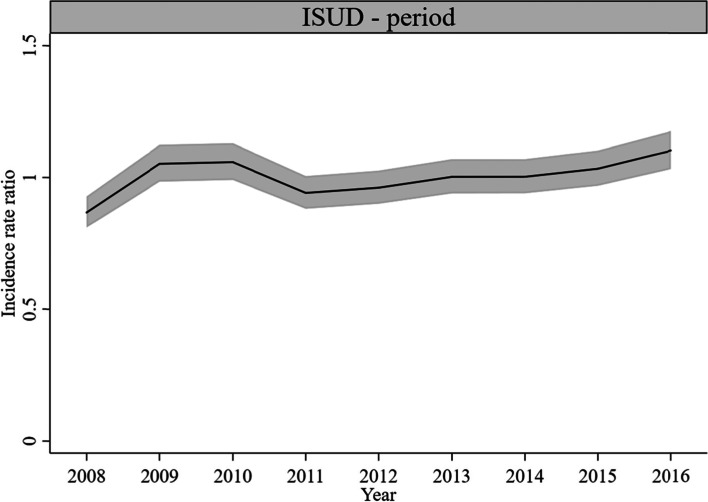
Period effects on ISUD-related utilization rate, 1-year period groups (*IRR* and 95% *CI*)

**Fig. 6:**
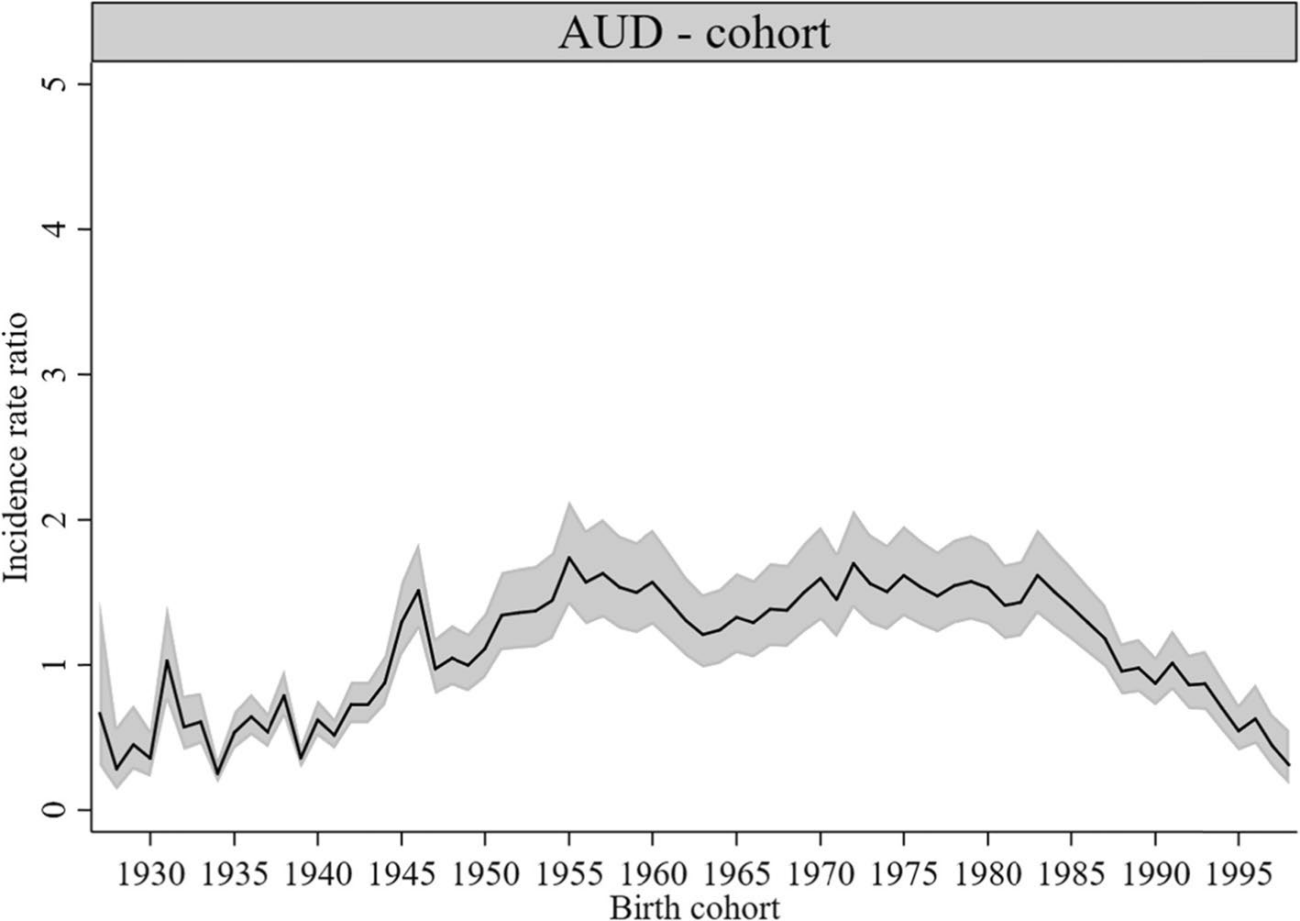
Cohort effects on AUD-related utilization rate, 1-year cohort groups (*IRR* and 95% *CI*)

**Fig. 7:**
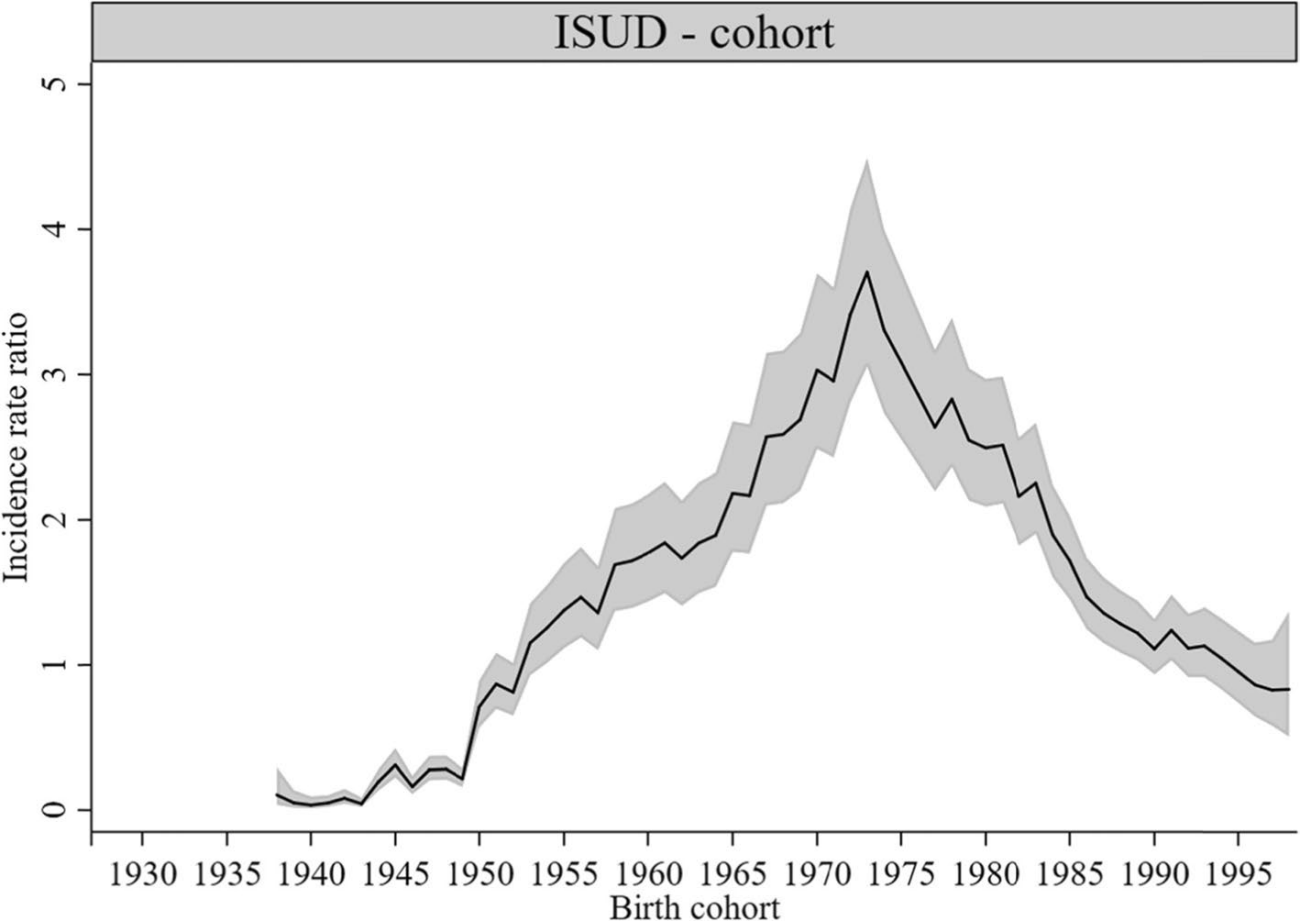
Cohort effects on ISUD-related utilization rate, 1-year cohort groups (*IRR* and 95% *CI*)

Figures 2–7 depict the results of the APC models and have different y-axis limits for easier illustration of the *IRR*. Most of the age effects were statistically significant for AUD- and ISUD-related utilization. For AUD-related utilization, a bi-modal pattern was observed (Fig. 2). The first peak in age effects was found in early adulthood and was followed by a subsequent decline. Starting at age 35 years, the rate increased again with a second and higher peak at age 50 years (*IRR* = 1.88, *CI* = 1.54, 2.30) and declined thereafter. The rate was lowest beginning from the age of 70 years onwards. ISUD-related utilization showed a nearly continuous decline in the *IRR* with increasing age (Fig. 3). A pronounced peak was found at the youngest ages (highest at age 18 years: *IRR* = 9.40, *CI* = 7.58, 11.66) followed by a steep decline until the mid-twenties. After this, the curve decreased more slowly. The lowest *IRR* was found at age 68 years (*IRR* = 0.17, *CI* = 0.11, 0.26).

#### Period effects

For AUD-related utilization, statistically significant period effects occurred in 2009 (*IRR* = 1.08, *CI* = 1.02, 1.14), 2012/2013 (*IRR* = 1.07, *CI* = 1.01, 1.13), 2015 (*IRR* = 0.92, *CI* = 0.87, 0.98), and 2016 (*IRR* = 0.91, *CI* = 0.86, 0.97) (Fig. 4). For ISUD-related utilization, there were statistically significant period effects in 2008 (*IRR* = 0.87, *CI* = 0.81, 0.93) and 2016 (*IRR* = 1.10, *CI* = 1.03, 1.18) (Fig. 5). The size of the effects was quite small.

#### Cohort effects

Cohort effects were statistically significant for most cohorts in both AUD- (Fig. 6) and ISUD-related utilization (Fig. 7). AUD-related utilization showed a plateau in birth cohorts from 1951 to 1986 (highest in cohort 1955: *IRR* = 1.74, *CI* = 1.42, 2.13). The earliest and latest birth cohorts showed lower *IRR* than the in-between cohorts. ISUD-related utilization increased from birth cohort 1954 to its peak in cohort 1973 (*IRR* = 3.70, *CI* = 3.06, 4.49). In later cohorts, the *IRR* declined again as rapidly as it had risen before. ISUD-related utilization was lowest in the cohorts born from 1938 to 1950. See Supplementary Table 1, Additional File 1 for the estimation of the *IRR*, 95% *CI*, and *P*-values for AUD- and ISUD-related utilization.

### Sensitivity analyses

The results of the APC models using 2-year intervals (SA1) are depicted in Supplementary Figs. 1–6 (Additional File 1) and show similar, but smoothed effects compared with the main analysis. The only exception is the year 2016 for AUD showing an increase in AUD-related utilization compared to the constant trend when using yearly data.

SA2 widely mirrored the results of the overall ISUD model (see Supplementary Figs. 7–15 and Supplementary Table 2, Additional File 1). Compared with the ISUD model, where age and cohort effects were mainly statistically significant, these effects were hardly significant for the distinct illicit substances. A detailed comparison revealed that age effects showed the same declining pattern from young to old age (minor differences: utilization for OUD and StiUD declined in the youngest ages but not continously, and CaUD-related utilization started at a considerably higher rate). The periodical pattern for ISUD-related utilization with a slight overall increase was also seen for the distinct illicit substances (minor difference: CaUD and StiUD although with a more pronounced increase). Cohort effects for OUD, CaUD, and StiUD ressembled those of ISUD-related utilization, with OUD and CaUD peaking at higher levels. CaUD- and StiUD-related utilization showed an additional peak in the late 40 s/early 50 s.

Regarding a change in the alpha level to 0.01, only a few of the estimated effects would not be statistically significant (SA3) (see Supplementary Table 3, Additional File 1).

## Discussion

Our APC analysis of trends in outpatient addiction care utilization in the general Berlin population revealed the highest age effects for AUD-related utilization in 18- to 19-year-old and in 39- to 59-year-old individuals. ISUD-related utilization declined almost continuously with increasing age. Period effects on trends in AUD- and ISUD-related utilization were quite small. Treatment utilization for AUD was highest in the cohorts born from 1951 to 1986, and ISUD-related utilization increased from the 1954 birth cohort to its peak in the 1973 birth cohort and declined with later cohorts.

### Age effects

AUD-related utilization showed a first and slight peak in 18- to 19-year-olds and a second one in middle age. Addiction prevention approaches are often targeted at younger age groups [14, 15], and intoxication-orientated drinking is a common drinking pattern in adolescence and early adulthood [16, 17]. This combination may increase the probability of early referral to addiction care when problematic alcohol use patterns occur. Furthermore, in young adulthood, living with parents and visiting educational institutions is frequent. Close bonds to family and pedagogical personnel may additionally enhance help-seeking in young adulthood [18].

In middle age, even higher AUD-related utilization rates were found than in young adulthood. Here, the onset of alcohol abuse occurred most likely some time ago [19], and the adverse effects of prolonged alcohol abuse might have become apparent. This increases the recognition of related problems, which in turn is associated with the onset of help-seeking [20–22]. In the oldest age groups, AUD-related utilization was lowest. As individuals with an alcohol abuse history have an increased risk of premature death [23], the proportion of individuals with AUD consequently decreases with older age [24]. This might resonate with a low utilization rate in older individuals. On the other hand, late onset AUD is an emerging issue [25], and insufficiently adapted diagnostic criteria for older age may lead to an underestimation of older individuals with AUD [26]. Together with older age-related barriers to making use of addiction care — such as denial of problematic use patterns [27], fear of stigma [28, 29], or lacking services tailored to this age group [30] — this may have contributed to the low AUD-related utilization rates in older age.

ISUD-related utilization decreased with increasing age. The pronounced peak in young adulthood may be explained by a similar combination as for AUD: frequent experimentation with illicit substance use in young adulthood [16], the main focus of addiction prevention on adolescents and young adults [14, 15], and the fact that young adults often have close bonds to their family and pedagogical personnel [18] probably enhance the likelihood of help-seeking. Furthermore, compared with AUD, the latency between onset of the disorder and treatment entry is known to be shorter for OUD, CaUD, and StiUD [31]. This may explain why the peaks in ISUD-related utilization were at younger ages than for AUD-related utilization. The reduced latency may result from a combination of a shorter transition from first cannabis or cocaine use to dependence [32], and illicit substance use being more frequently associated with a faster progression into marginalization [23, 33, 34]. These factors most likely increase the recognition of related problems which, as mentioned earlier, is linked to help-seeking [20, 22]. ISUD-related utilization was lowest in the oldest age groups from age 54 onwards. Similar to AUD, premature death is common in individuals with ISUD [23], and the prevalence of ISUD is low in older age groups [35], possibly contributing to the low ISUD-related utilization rates in older age.

### Period effects

When controlled for age and cohort, period effects on trends in AUD-related utilization were quite small. Over the relatively short period of 9 years, no major legislative and economic changes took place that could have affected access requirements and the use of addiction care. It is known that only a minority of individuals with AUD seek help [36, 37]. In light of the slightly decreasing prevalence of AUD in the general male population and the rather stable AUD prevalence in females between 2006 and 2018 [38], the small declining trend in AUD-related utilization suggests a rather stable reach of individuals affected with AUD.

The ISUD-related period effects were small as well. A slight and singular significant increase was observed in 2016, which may correspond with the implementation of interventions targeting cannabis users [39]. It is important to note that our results mirror the pooled effects of ISUD-related utilization and that, in our sample, OUD-related utilization considerably outweighs CaUD-related utilization. A review of studies on illicit substance use in German-speaking countries reported a decreasing tendency for heroin use and a high number of “old users” in treatment [40]. Studies on ISUD in Germany indicate a rather stable trend of the number of individuals with OUD [7] and slightly increasing trends in the prevalence of CaUD and StiUD over the last 20 years [38]. This trend was supported by our sensitivity analyses. Overall, the rather similar period effects of trends in outpatient addiction care utilization for OUD, CaUD, and StiUD underscore the notion that, apart from changes in the affected populations, no major changes in outpatient addiction care utilization could be observed for ISUD.

### Cohort effects

The earliest birth cohorts born between 1928 and 1943 showed relatively low AUD-related utilization rates, whereas the highest rates were found in the cohorts born between 1951 and 1986. Previous findings on the extent of alcohol use problems indicate higher alcohol use rates in the so-called baby boomer generation compared with earlier cohorts [41, 42]. In later cohorts born from 1994 onwards, AUD-related utilization was relatively low. These changes may reflect the observed lower drinking prevalence rates in more recent cohorts compared with cohorts born before 1970 [17]. As one possible explanation for the decline in youth drinking in recent years, the “devaluation” of alcohol has been discussed, emerging from a combination of major changes in family relationships, gender identity, and lifestyle as well as social reactions to the negative effects of alcohol use [43]. This may have reduced the number of individuals needing treatment for AUD in the respective cohorts. Apparently, the cohort effects in treatment utilization primarily follow changes in drinking behavior and thus mirror changes in treatment need rather than changes in the provision of cohort-specific outpatient addiction care.

The lowest rates regarding ISUD-related utilization were found in the earliest analyzed cohorts. There is evidence that limited health care provision for illicit substance abusers in the past has reduced the survival odds of these groups, resulting in diminished numbers of individuals with ISUD in earlier cohorts [44]. Our finding of the highest ISUD-related utilization rates in the cohorts born between 1954 and 1989 appears to correspond with the above-mentioned increase in baby boomers with substance use problems including illicit substances [41, 42]. This is particularly true for the cohorts born between 1965 and 1983, largely corresponding with the drug wave of the late 1960s and 1970s [47]. The plateau also continued into the cohorts born after the baby boomers until about the late 1980s. An earlier study of Berlin outpatient addiction care has already indicated that cohorts born after the baby boomers are more likely to seek help for primary ISUD [3]. This evidence is now substantiated with a more comprehensive methodological approach and for a wider age range.

### Limitation and strengths

Our study is not without limitations. First, as different APC effects might occur in particularly rural German regions, the generalizability to the whole of Germany should be handled with care. Second, the mode of data collection was uniform for the comparatively short timeframe of 9 years. Hence, long-term trends could not be analyzed, and more substantial period effects might occur in a longer period of time. This emphasizes the need to maintain comparable data collection modes. Third, owing to challenges in detecting multiple individual counts in half the cases in 2010, the number of individuals seeking help was somewhat overestimated in 2010. On average, 1.2 episodes per individual were found for the cases with an undamaged identifier variable. Excluding the cases with a damaged identifier variable would have caused greater bias (underestimation) than accepting the presumable low overestimation. This seems to be negligible as the period trends in 2010 did not turn upwards.

The analysis of APC effects on trends in AUD- and ISUD-related outpatient addiction care utilization in a German setting is an innovative study approach. The sample covers almost all individuals using outpatient addiction care in Berlin, reducing the risk of selection bias. To investigate the consistency of the APC effects in the compound measure of ISUD, we separated this group into the most prevalent diagnoses. These analyses largely confirmed the results of the pooled ISUD analysis. The overall APC effects were rather invariant to models using 2-year intervals instead of single-years or an application of an alpha level of 0.01.

## Conclusions

Age and cohort were the strongest drivers of trends in AUD- and ISUD-related outpatient addiction care utilization in the general Berlin population. The lack of substantial period effects might result from the short period time available. Considering delayed help-seeking, the finding of the highest AUD-related utilization rates in middle age indicates the need for interventions facilitating treatment uptake at an early stage of AUD development. The constant period-related utilization rates suggest a stable attainment rate of addiction care. Considering changes in the affected populations of individuals with AUD or ISUD and low utilization rates for AUD and ISUD per se [36, 37], enhancing addiction care utilization appears to be essential for reducing substance-related morbidity and mortality [45, 46]. The highest utilization rates for AUD and ISUD in the baby boomer generation and following cohorts underline an increased demand for addiction care and imply that substance abuse in youth and young adulthood translates into demand for addiction care at older ages.

## Supplementary Information

**Additional file 1.** Supplementary Table for the estimation of the IRR, 95% *CI*, and *P*-values for AUD- and ISUD-related utilization and Supplementary Figures and Tables regarding the sensitivity analyses SA1-SA3

## Data Availability

The datasets generated and analyzed during the current study are not publicly available due to data protection requirements but are available from the corresponding author on reasonable request. The size of the general Berlin population was taken from the German Federal Statistical Office (9) and is available in the Genesis-Online Database repository, https://www-genesis.destatis.de/genesis/online.

## Abbreviations

APC: Age, period, and cohort
AUD: Alcohol use disorders
CaUD: Cannabis use disorders
CI: Confidence interval
IRR: Incidence rate ratio
ISUD: Illicit substances use disorders
OUD: Opioids use disorders
SA1: Sensitivity analysis 1, APC models with 2-year interval
SA2: Sensitivity analysis 2, targeted at the utilization rate of distinct illicit substances
SA3: Sensitivity analysis 3, using of an alpha level of 0.01
StiUD: Stimulants/cocaine use disorders
