# Supplementary material for: Age, period, and cohort effects on trends in outpatient addiction care utilization in the general Berlin population from 2008 to 2016
Source: BMC Public Health. 2022 Feb 15;22:320. doi: 10.1186/s12889-022-12744-6 (PMC8848644; doi:10.1186/s12889-022-12744-6)
Supplement: Supplementary file 1 — Additional file 1. Supplementary Table for the estimation of the IRR, 95% CI, and P-values for AUD- and ISUD-related utilization and Supplementary Figures and Tables regarding the sensitivity analyses SA1-SA3 [file 12889_2022_12744_MOESM1_ESM.docx]

Supplementary Table 1. Negative binomial APC models on AUD- and ISUD-related utilization rate

|  | AUD | |  | ISUD | |
| --- | --- | --- | --- | --- | --- |
|  | *IRR* (95% *CI*) | *P* |  | *IRR* (95% *CI*) | *P* |
| Intercept | 0.00 (0.00, 0.00) | <0.001 |  | 0.00 (0.00, 0.00) | <0.001 |
| Age |  |  |  |  |  |
| 18 | 1.51 (1.20, 1.91) | <0.001 |  | 9.40 (7.58, 11.66) | <0.001 |
| 19 | 1.43 (1.15, 1.78) | 0.001 |  | 9.18 (7.50, 11.25) | <0.001 |
| 20 | 1.31 (1.06, 1.62) | 0.012 |  | 7.96 (6.55, 9.68) | <0.001 |
| 21 | 1.22 (0.99, 1.49) | 0.061 |  | 6.77 (5.60, 8.19) | <0.001 |
| 22 | 0.97 (0.80, 1.19) | 0.791 |  | 5.52 (4.59, 6.64) | <0.001 |
| 23 | 0.88 (0.73, 1.07) | 0.205 |  | 4.55 (3.80, 5.45) | <0.001 |
| 24 | 0.82 (0.68, 0.99) | 0.039 |  | 3.80 (3.19, 4.54) | <0.001 |
| 25 | 0.80 (0.67, 0.97) | 0.021 |  | 3.43 (2.89, 4.08) | <0.001 |
| 26 | 0.80 (0.66, 0.96) | 0.015 |  | 2.86 (2.41, 3.40) | <0.001 |
| 27 | 0.72 (0.60, 0.87) | 0.001 |  | 2.54 (2.14, 3.02) | <0.001 |
| 28 | 0.80 (0.67, 0.96) | 0.017 |  | 2.37 (2.00, 2.81) | <0.001 |
| 29 | 0.77 (0.64, 0.92) | 0.004 |  | 2.12 (1.79, 2.51) | <0.001 |
| 30 | 0.85 (0.71, 1.02) | 0.081 |  | 1.96 (1.66, 2.33) | <0.001 |
| 31 | 0.86 (0.72, 1.03) | 0.106 |  | 1.83 (1.54, 2.17) | <0.001 |
| 32 | 0.95 (0.79, 1.13) | 0.556 |  | 1.79 (1.50, 2.12) | <0.001 |
| 33 | 1.01 (0.85, 1.22) | 0.878 |  | 1.68 (1.41, 2.00) | <0.001 |
| 34 | 1.06 (0.88, 1.27) | 0.551 |  | 1.52 (1.27, 1.81) | <0.001 |
| 35 | 1.22 (1.02, 1.47) | 0.032 |  | 1.49 (1.24, 1.77) | <0.001 |
| 36 | 1.26 (1.05, 1.52) | 0.014 |  | 1.40 (1.17, 1.67) | <0.001 |
| 37 | 1.33 (1.11, 1.61) | 0.002 |  | 1.36 (1.13, 1.62) | 0.001 |
| 38 | 1.31 (1.08, 1.58) | 0.005 |  | 1.27 (1.06, 1.52) | 0.010 |
| 39 | 1.58 (1.31, 1.91) | <0.001 |  | 1.13 (0.94, 1.36) | 0.184 |
| 40 | 1.49 (1.23, 1.81) | <0.001 |  | 1.20 (0.99, 1.44) | 0.057 |
| 41 | 1.61 (1.33, 1.95) | <0.001 |  | 1.04 (0.86, 1.25) | 0.670 |
| 42 | 1.66 (1.37, 2.02) | <0.001 |  | 1.01 (0.84, 1.22) | 0.934 |
| 43 | 1.67 (1.38, 2.03) | <0.001 |  | 0.94 (0.79, 1.13) | 0.519 |
| 44 | 1.81 (1.48, 2.20) | <0.001 |  | 0.90 (0.75, 1.09) | 0.300 |
| 45 | 1.75 (1.44, 2.13) | <0.001 |  | 0.86 (0.71, 1.04) | 0.110 |
| 46 | 1.77 (1.45, 2.16) | <0.001 |  | 0.79 (0.65, 0.95) | 0.014 |
| 47 | 1.77 (1.45, 2.16) | <0.001 |  | 0.75 (0.62, 0.91) | 0.004 |
| 48 | 1.82 (1.49, 2.22) | <0.001 |  | 0.71 (0.59, 0.86) | 0.001 |
| 49 | 1.84 (1.51, 2.25) | <0.001 |  | 0.68 (0.57, 0.83) | <0.001 |
| 50 | 1.88 (1.54, 2.30) | <0.001 |  | 0.63 (0.52, 0.77) | <0.001 |
| 51 | 1.86 (1.52, 2.27) | <0.001 |  | 0.65 (0.54, 0.79) | <0.001 |
| 52 | 1.81 (1.49, 2.22) | <0.001 |  | 0.53 (0.43, 0.64) | <0.001 |
| 53 | 1.75 (1.43, 2.14) | <0.001 |  | 0.55 (0.46, 0.67) | <0.001 |
| 54 | 1.68 (1.38, 2.05) | <0.001 |  | 0.48 (0.40, 0.59) | <0.001 |
| 55 | 1.71 (1.40, 2.08) | <0.001 |  | 0.49 (0.40, 0.60) | <0.001 |
| 56 | 1.64 (1.35, 2.00) | <0.001 |  | 0.49 (0.40, 0.59) | <0.001 |
| 57 | 1.61 (1.32, 1.96) | <0.001 |  | 0.41 (0.34, 0.50) | <0.001 |
| 58 | 1.54 (1.26, 1.87) | <0.001 |  | 0.42 (0.35, 0.51) | <0.001 |
| 59 | 1.45 (1.19, 1.76) | <0.001 |  | 0.38 (0.31, 0.47) | <0.001 |
| 60 | 1.38 (1.14, 1.67) | 0.001 |  | 0.37 (0.30, 0.46) | <0.001 |
| 61 | 1.34 (1.10, 1.62) | 0.003 |  | 0.32 (0.25, 0.39) | <0.001 |
| 62 | 1.30 (1.07, 1.57) | 0.007 |  | 0.38 (0.30, 0.48) | <0.001 |
| 63 | 1.16 (0.96, 1.40) | 0.128 |  | 0.33 (0.26, 0.42) | <0.001 |
| 64 | 1.07 (0.88, 1.29) | 0.487 |  | 0.24 (0.18, 0.32) | <0.001 |
| 65 | 0.99 (0.82, 1.20) | 0.937 |  | 0.26 (0.20, 0.35) | <0.001 |
| 66 | 0.93 (0.77, 1.12) | 0.434 |  | 0.21 (0.15, 0.29) | <0.001 |
| 67 | 0.85 (0.71, 1.03) | 0.103 |  | 0.34 (0.25, 0.47) | <0.001 |
| 68 | 0.82 (0.67, 0.99) | 0.036 |  | 0.17 (0.11, 0.26) | <0.001 |
| 69 | 0.76 (0.63, 0.92) | 0.005 |  | 0.25 (0.16, 0.37) | <0.001 |
| 70 | 0.61 (0.51, 0.75) | <0.001 |  | 0.42 (0.28, 0.63) | <0.001 |
| 71 | 0.66 (0.54, 0.80) | <0.001 |  |  |  |
| 72 | 0.55 (0.45, 0.67) | <0.001 |  |  |  |
| 73 | 0.39 (0.32, 0.48) | <0.001 |  |  |  |
| 74 | 0.42 (0.34, 0.52) | <0.001 |  |  |  |
| 75 | 0.47 (0.38, 0.58) | <0.001 |  |  |  |
| 76 | 0.27 (0.21, 0.35) | <0.001 |  |  |  |
| 77 | 0.23 (0.17, 0.29) | <0.001 |  |  |  |
| 78 | 0.31 (0.24, 0.40) | <0.001 |  |  |  |
| 79 | 0.36 (0.28, 0.47) | <0.001 |  |  |  |
| 80 | 0.22 (0.16, 0.30) | <0.001 |  |  |  |
| 81 | 0.28 (0.20, 0.39) | <0.001 |  |  |  |
| Period |  |  |  |  |  |
| 2008 | 0.95 (0.89, 1.01) | 0.079 |  | 0.87 (0.81, 0.93) | <0.001 |
| 2009 | 1.08 (1.02, 1.14) | 0.011 |  | 1.05 (0.98, 1.12) | 0.134 |
| 2010 | 1.02 (0.96, 1.08) | 0.545 |  | 1.06 (0.99, 1.13) | 0.090 |
| 2011 | 1.01 (0.96, 1.08) | 0.624 |  | 0.94 (0.88, 1.01) | 0.071 |
| 2012 | 1.07 (1.01, 1.13) | 0.024 |  | 0.96 (0.90, 1.03) | 0.233 |
| 2013 | 1.07 (1.01, 1.13) | 0.025 |  | 1.00 (0.94, 1.07) | 0.937 |
| 2014 | 0.98 (0.92, 1.04) | 0.486 |  | 1.00 (0.94, 1.07) | 0.932 |
| 2015 | 0.92 (0.87, 0.98) | 0.009 |  | 1.03 (0.97, 1.10) | 0.318 |
| 2016 | 0.91 (0.86, 0.97) | 0.003 |  | 1.10 (1.03, 1.18) | 0.004 |
| Cohort |  |  |  |  |  |
| 1927 | 0.66 (0.31, 1.44) | 0.300 |  |  |  |
| 1928 | 0.28 (0.14, 0.57) | <0.001 |  |  |  |
| 1929 | 0.45 (0.28, 0.72) | 0.001 |  |  |  |
| 1930 | 0.35 (0.23, 0.55) | <0.001 |  |  |  |
| 1931 | 1.03 (0.75, 1.41) | 0.860 |  |  |  |
| 1932 | 0.57 (0.41, 0.79) | 0.001 |  |  |  |
| 1933 | 0.61 (0.46, 0.81) | 0.001 |  |  |  |
| 1934 | 0.25 (0.19, 0.34) | <0.001 |  |  |  |
| 1935 | 0.53 (0.42, 0.68) | <0.001 |  |  |  |
| 1936 | 0.64 (0.51, 0.80) | <0.001 |  |  |  |
| 1937 | 0.54 (0.43, 0.67) | <0.001 |  |  |  |
| 1938 | 0.79 (0.64, 0.97) | 0.023 |  | 0.10 (0.04, 0.29) | <0.001 |
| 1939 | 0.36 (0.29, 0.45) | <0.001 |  | 0.05 (0.02, 0.14) | <0.001 |
| 1940 | 0.62 (0.51, 0.76) | <0.001 |  | 0.04 (0.01, 0.09) | <0.001 |
| 1941 | 0.51 (0.42, 0.63) | <0.001 |  | 0.05 (0.02, 0.10) | <0.001 |
| 1942 | 0.73 (0.60, 0.88) | 0.001 |  | 0.81 (0.05, 0.15) | <0.001 |
| 1943 | 0.73 (0.60, 0.88) | 0.001 |  | 0.04 (0.02, 0.09) | <0.001 |
| 1944 | 0.88 (0.72, 1.06) | 0.183 |  | 0.19 (0.14, 0.28) | <0.001 |
| 1945 | 1.30 (1.07, 1.57) | 0.009 |  | 0.31 (0.26, 0.42) | <0.001 |
| 1946 | 1.51 (1.25, 1.84) | <0.001 |  | 0.16 (0.11, 0.24) | <0.001 |
| 1947 | 0.97 (0.80, 1.18) | 0.780 |  | 0.28 (0.21, 0.37) | <0.001 |
| 1948 | 1.05 (0.86, 1.27) | 0.652 |  | 0.28 (0.21, 0.37) | <0.001 |
| 1949 | 1.00 (0.82, 1.22) | 0.979 |  | 0.22 (0.16, 0.29) | <0.001 |
| 1950 | 1.11 (0.91, 1.36) | 0.296 |  | 0.71 (0.57, 0.89) | 0.003 |
| 1951 | 1.34 (1.10, 1.64) | 0.004 |  | 0.87 (0.70, 1.08) | 0.211 |
| 1952 | 1.36 (1.11, 1.66) | 0.003 |  | 0.81 (0.65, 1.01) | 0.065 |
| 1953 | 1.37 (1.12, 1.68) | 0.002 |  | 1.15 (0.93, 1.42) | 0.198 |
| 1954 | 1.45 (1.18, 1.77) | <0.001 |  | 1.25 (1.01, 1.55) | 0.036 |
| 1955 | 1.74 (1.42, 2.13) | <0.001 |  | 1.38 (1.12, 1.70) | 0.003 |
| 1956 | 1.57 (1.28, 1.93) | <0.001 |  | 1.47 (1.19, 1.81) | <0.001 |
| 1957 | 1.63 (1.33, 2.00) | <0.001 |  | 1.36 (1.10, 1.68) | 0.004 |
| 1958 | 1.54 (1.25, 1.89) | <0.001 |  | 1.69 (1.37, 2.08) | <0.001 |
| 1959 | 1.50 (1.22, 1.85) | <0.001 |  | 1.72 (1.39, 2.11) | <0.001 |
| 1960 | 1.57 (1.28, 1.93) | <0.001 |  | 1.77 (1.44, 2.18) | <0.001 |
| 1961 | 1.44 (1.17, 1.77) | 0.001 |  | 1.84 (1.50, 2.26) | <0.001 |
| 1962 | 1.30 (1.06, 1.61) | 0.012 |  | 1.73 (1.41, 2.13) | <0.001 |
| 1963 | 1.21 (0.98, 1.49) | 0.073 |  | 1.84 (1.50, 2.56) | <0.001 |
| 1964 | 1.24 (1.01, 1.53) | 0.042 |  | 1.89 (1.54, 2.32) | <0.001 |
| 1965 | 1.33 (1.08, 1.63) | 0.007 |  | 2.18 (1.78, 2.68) | <0.001 |
| 1966 | 1.29 (1.05, 1.59) | 0.015 |  | 2.17 (1.77, 2.66) | <0.001 |
| 1967 | 1.39 (1.13, 1.70) | 0.002 |  | 2.57 (2.10, 3.15) | <0.001 |
| 1968 | 1.38 (1.12, 1.69) | 0.002 |  | 2.59 (2.12, 3.16) | <0.001 |
| 1969 | 1.50 (1.22, 1.83) | <0.001 |  | 2.69 (2.20, 3.29) | <0.001 |
| 1970 | 1.60 (1.31, 1.95) | <0.001 |  | 3.03 (2.49, 3.70) | <0.001 |
| 1971 | 1.45 (1.19, 1.77) | <0.001 |  | 2.96 (2.43, 3.60) | <0.001 |
| 1972 | 1.70 (1.39, 2.07) | <0.001 |  | 3.41 (2.81, 4.14) | <0.001 |
| 1973 | 1.56 (1.28, 1.90) | <0.001 |  | 3.70 (3.06, 4.49) | <0.001 |
| 1974 | 1.51 (1.24, 1.83) | <0.001 |  | 3.30 (2.73, 4.00) | <0.001 |
| 1975 | 1.62 (1.34, 1.96) | <0.001 |  | 3.08 (2.56, 3.72) | <0.001 |
| 1976 | 1.54 (1.27, 1.86) | <0.001 |  | 2.86 (2.38, 3.44) | <0.001 |
| 1977 | 1.48 (1.22, 1.78) | <0.001 |  | 2.64 (2.20, 3.17) | <0.001 |
| 1978 | 1.55 (1.29, 1.86) | <0.001 |  | 2.83 (2.36, 3.39) | <0.001 |
| 1979 | 1.58 (1.31, 1.89) | <0.001 |  | 2.55 (2.13, 3.04) | <0.001 |
| 1980 | 1.53 (1.28, 1.84) | <0.001 |  | 2.49 (2.09, 2.97) | <0.001 |
| 1981 | 1.41 (1.18, 1.69) | <0.001 |  | 2.51 (2.11, 2.99) | <0.001 |
| 1982 | 1.43 (1.20, 1.71) | <0.001 |  | 2.16 (1.82, 2.56) | <0.001 |
| 1983 | 1.62 (1.35, 1.93) | <0.001 |  | 2.25 (1.90, 2.67) | <0.001 |
| 1984 | 1.50 (1.26, 1.80) | <0.001 |  | 1.89 (1.60, 2.24) | <0.001 |
| 1985 | 1.40 (1.17, 1.68) | <0.001 |  | 1.72 (1.45, 2.03) | <0.001 |
| 1986 | 1.29 (1.08, 1.54) | 0.005 |  | 1.47 (1.25, 1.74) | <0.001 |
| 1987 | 1.18 (0.99, 1.42) | 0.071 |  | 1.36 (1.15, 1.60) | <0.001 |
| 1988 | 0.96 (0.80, 1.15) | 0.631 |  | 1.28 (1.08, 1.52) | 0.004 |
| 1989 | 0.98 (0.81, 1.18) | 0.820 |  | 1.22 (1.03, 1.44) | 0.021 |
| 1990 | 0.87 (0.72, 1.05) | 0.158 |  | 1.11 (0.94, 1.32) | 0.233 |
| 1991 | 1.01 (0.83, 1.24) | 0.901 |  | 1.24 (1.03, 1.49) | 0.022 |
| 1992 | 0.86 (0.69, 1.07) | 0.179 |  | 1.11 (0.92, 1.35) | 0.279 |
| 1993 | 0.87 (0.69, 1.10) | 0.241 |  | 1.13 (0.92, 1.40) | 0.252 |
| 1994 | 0.70 (0.55, 0.91) | 0.007 |  | 1.05 (0.83, 1.32) | 0.696 |
| 1995 | 0.54 (0.41, 0.72) | <0.001 |  | 0.95 (0.74, 1.23) | 0.724 |
| 1996 | 0.63 (0.45, 0.87) | 0.005 |  | 0.86 (0.64, 1.15) | 0.318 |
| 1997 | 0.44 (0.30, 0.66) | <0.001 |  | 0.83 (0.58, 1.17) | 0.285 |
| 1998 | 0.31 (0.17, 0.55) | <0.001 |  | 0.83 (0.51, 1.36) | 0.464 |
| Observations | 576 |  |  | 477 |  |
| BIC | –2,734.56 |  |  | –2,183.07 |  |

*Note*. APC = age, period, and cohort; AUD = alcohol use disorders; ISUD = illicit substances use disorders; *IRR* = incidence rate ratio; *CI* = confidence interval; BIC = Bayesian information criterion.


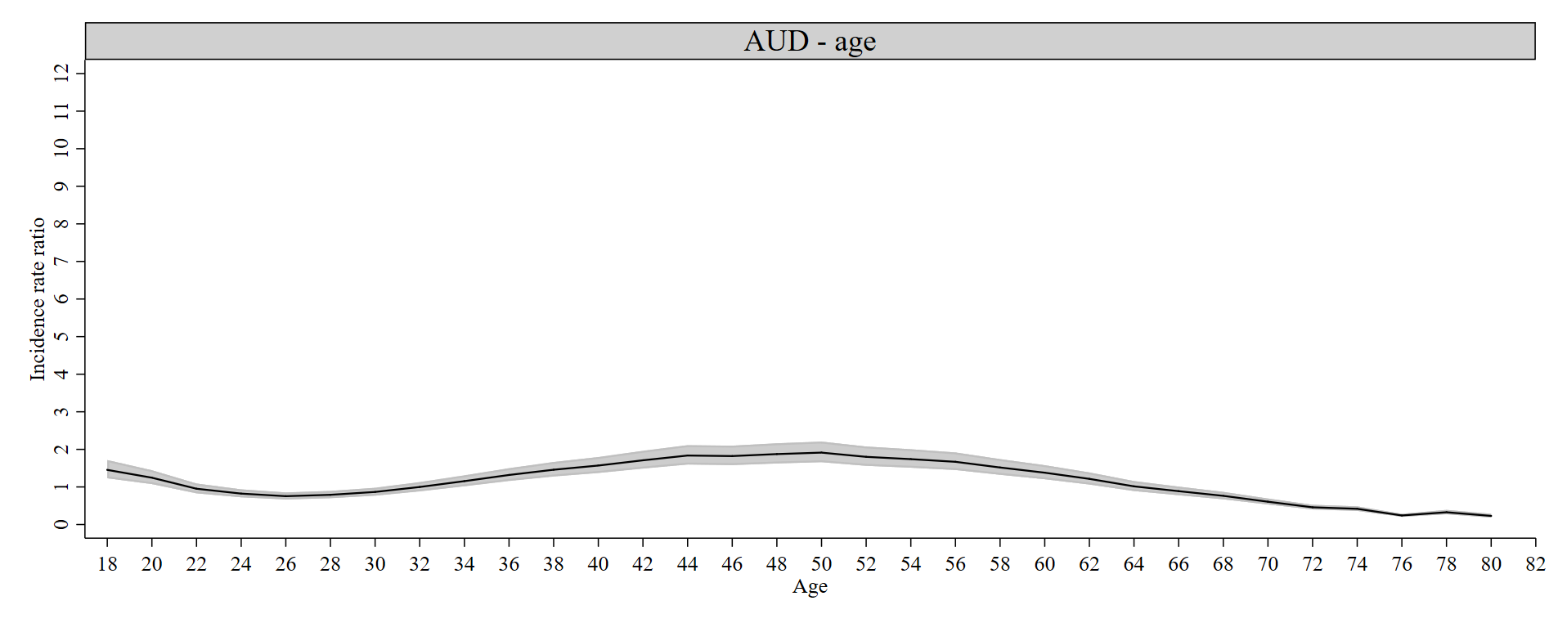


Supplementary Figure 1. Age effects on AUD-related utilization rate, 2-year age groups (*IRR*)


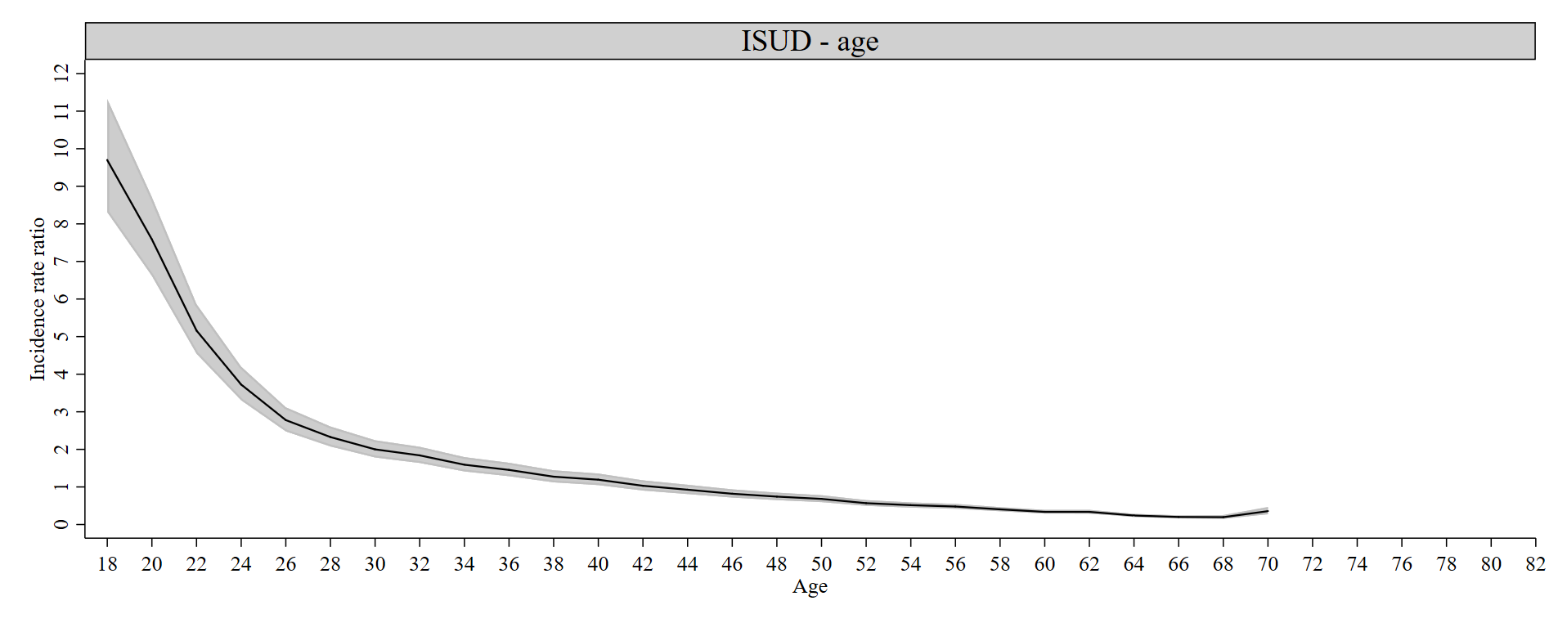


Supplementary Figure 2. Age effects on ISUD-related utilization rate, 2-year age groups (*IRR*)


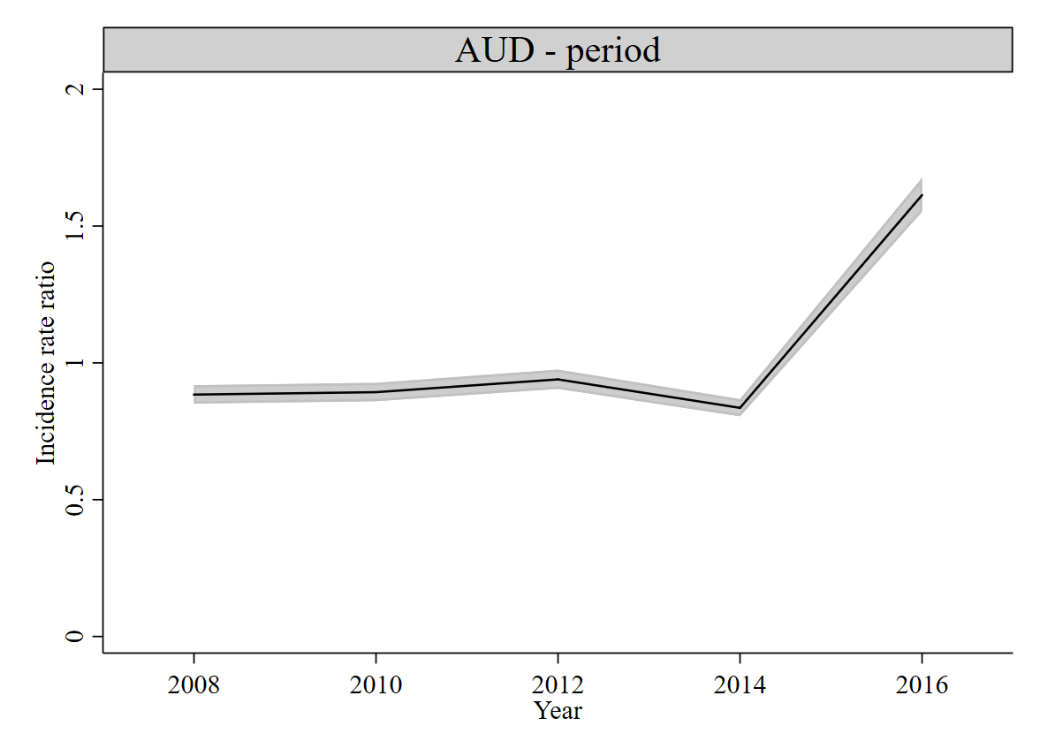


Supplementary Figure 3. Period effects on AUD-related utilization rate, 2-year period groups (*IRR*)


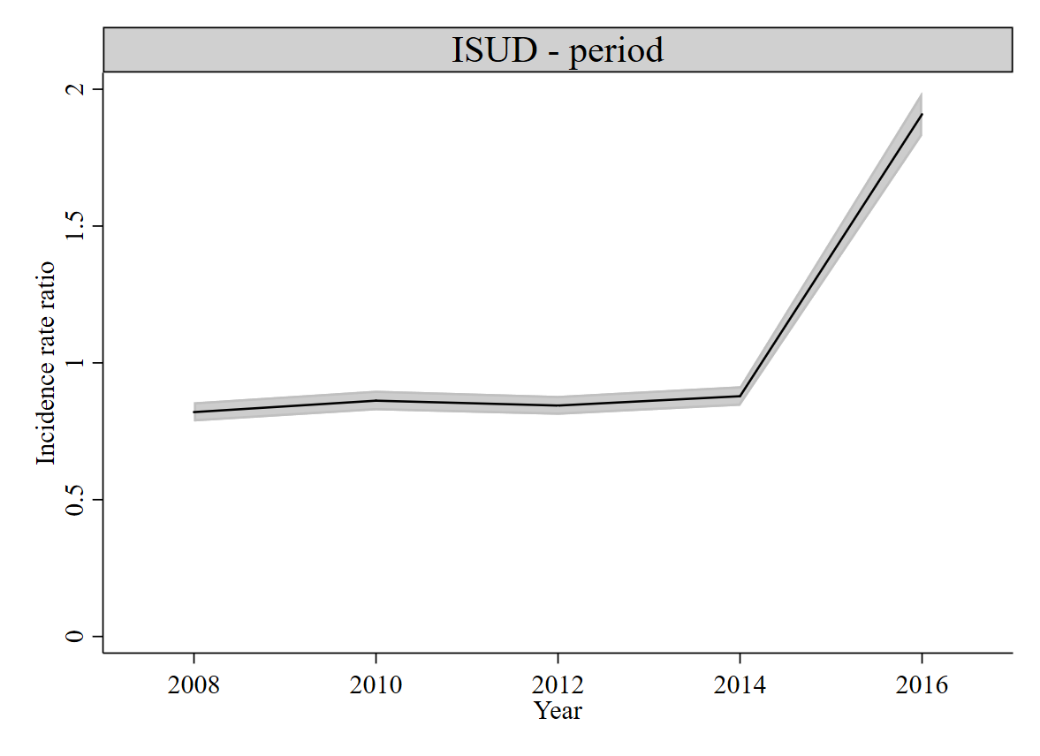


Supplementary Figure 4. Period effects on ISUD-related utilization rate, 2-year period groups (*IRR*)


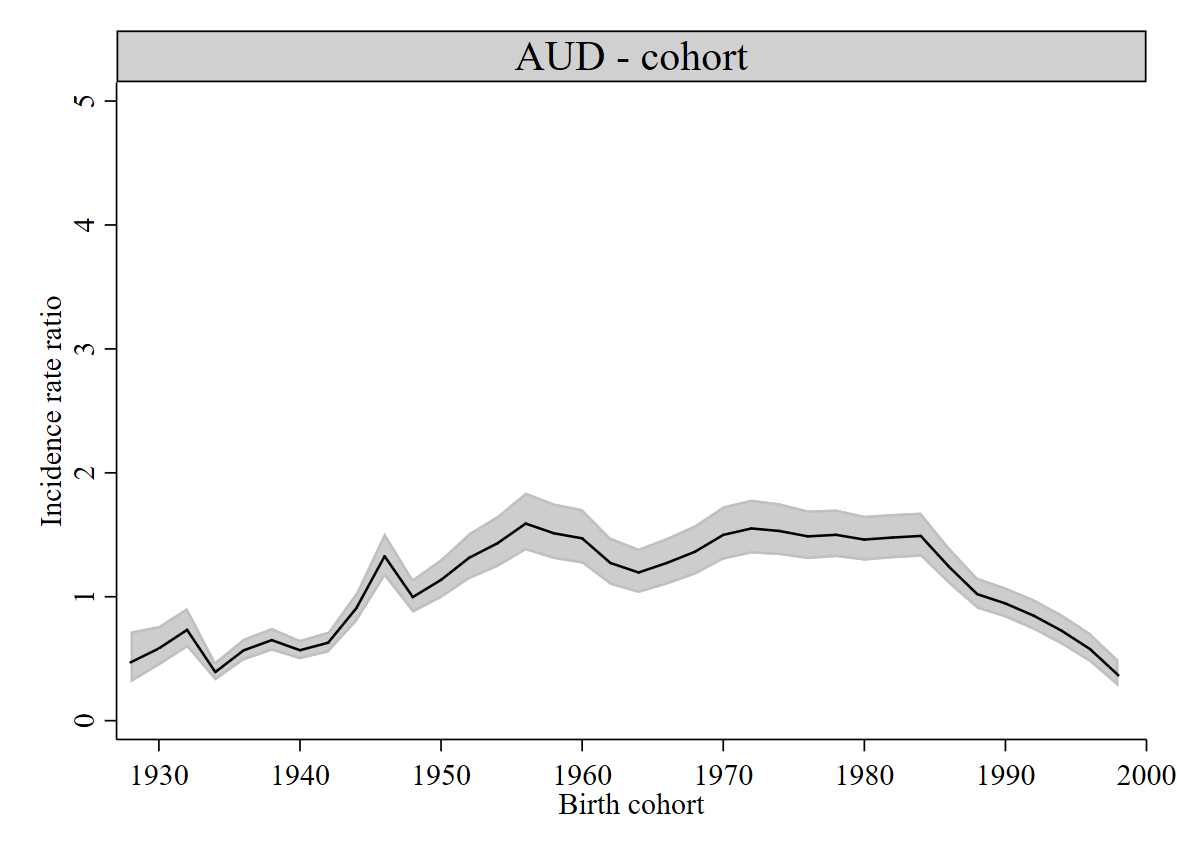


Supplementary Figure 5. Cohort effects on AUD-related utilization rate, 2-year cohort groups (*IRR*)


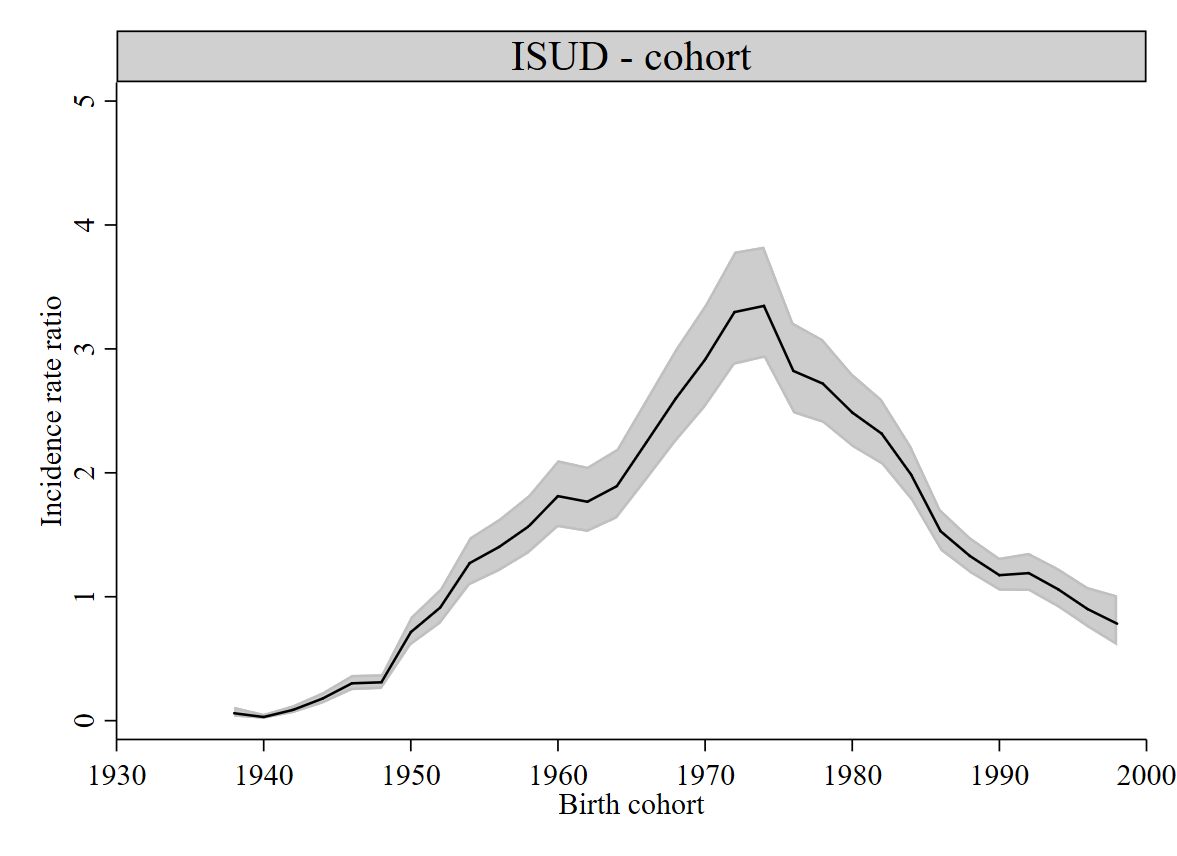


Supplementary Figure 6. Cohort effects on ISUD-related utilization rate, 2-year cohort groups (*IRR*)


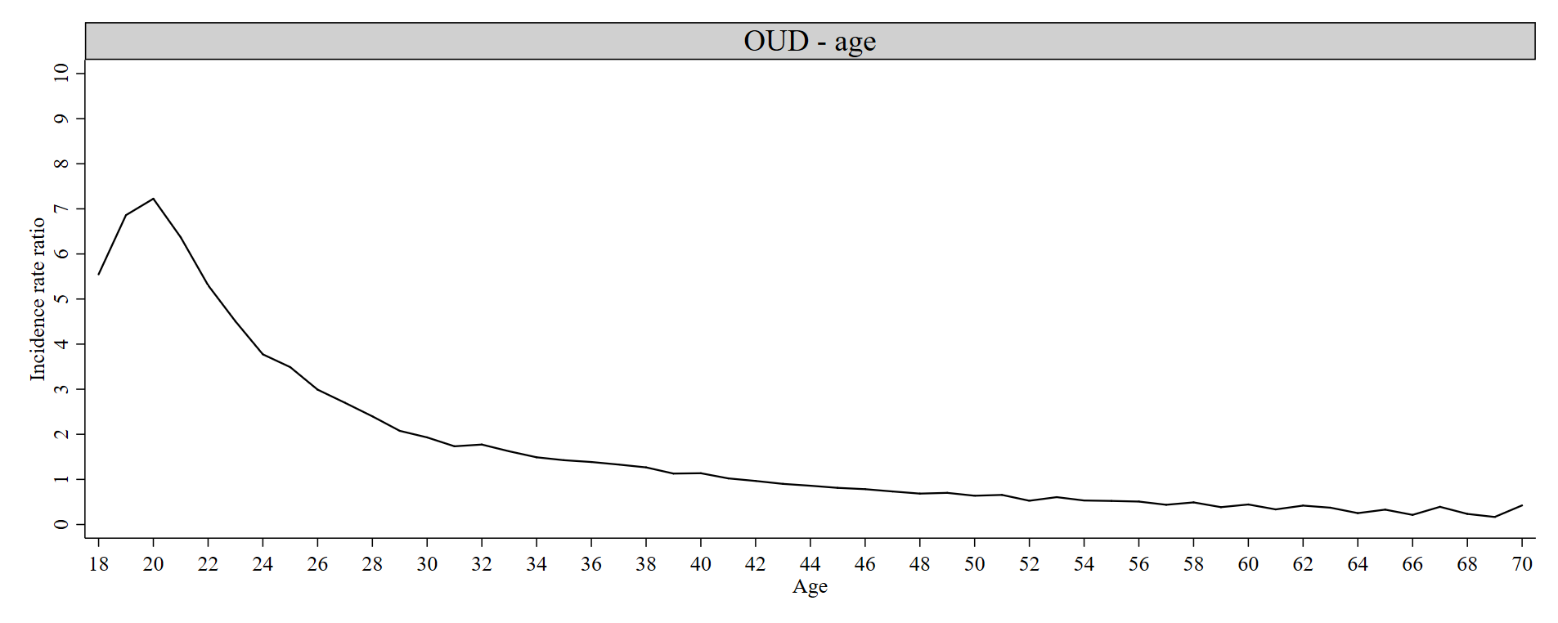


Supplementary Figure 7. Age effects on OUD-related utilization rate, 1-year age groups (*IRR*)


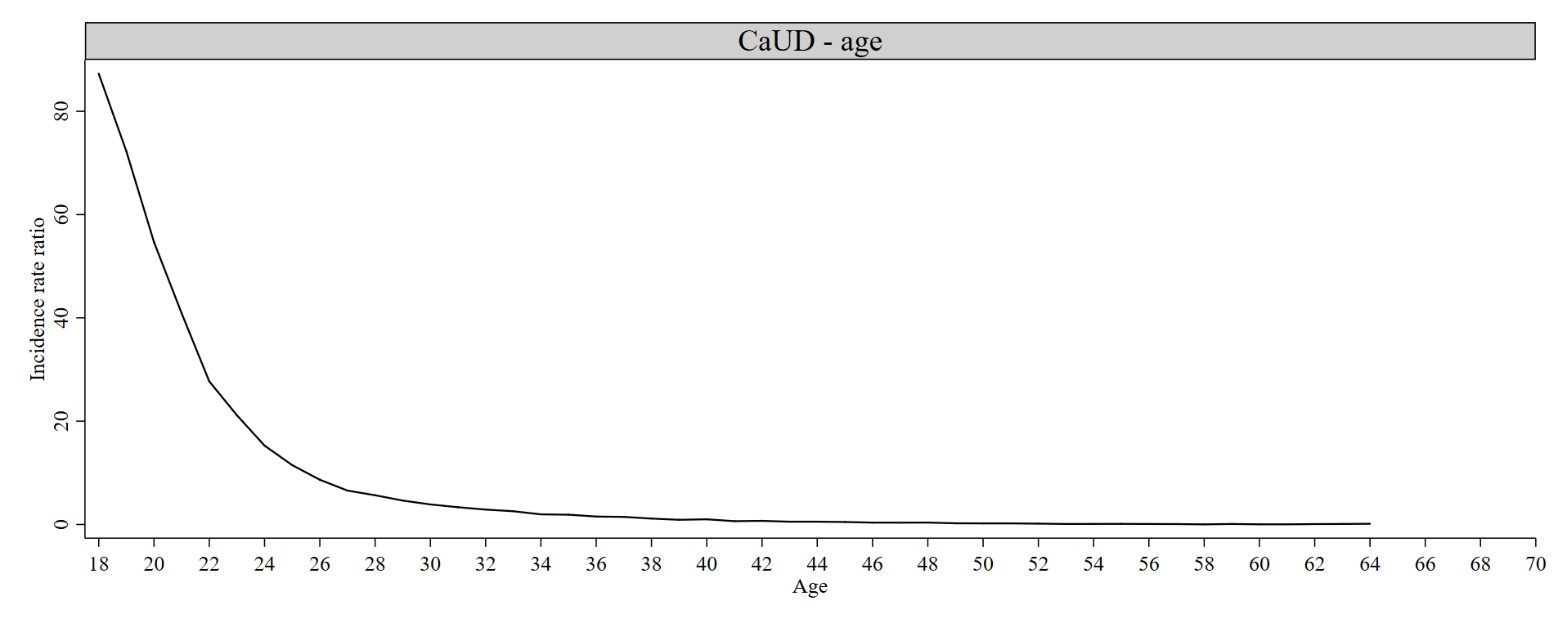


Supplementary Figure 8. Age effects on CaUD-related utilization rate, 1-year age groups (*IRR*)


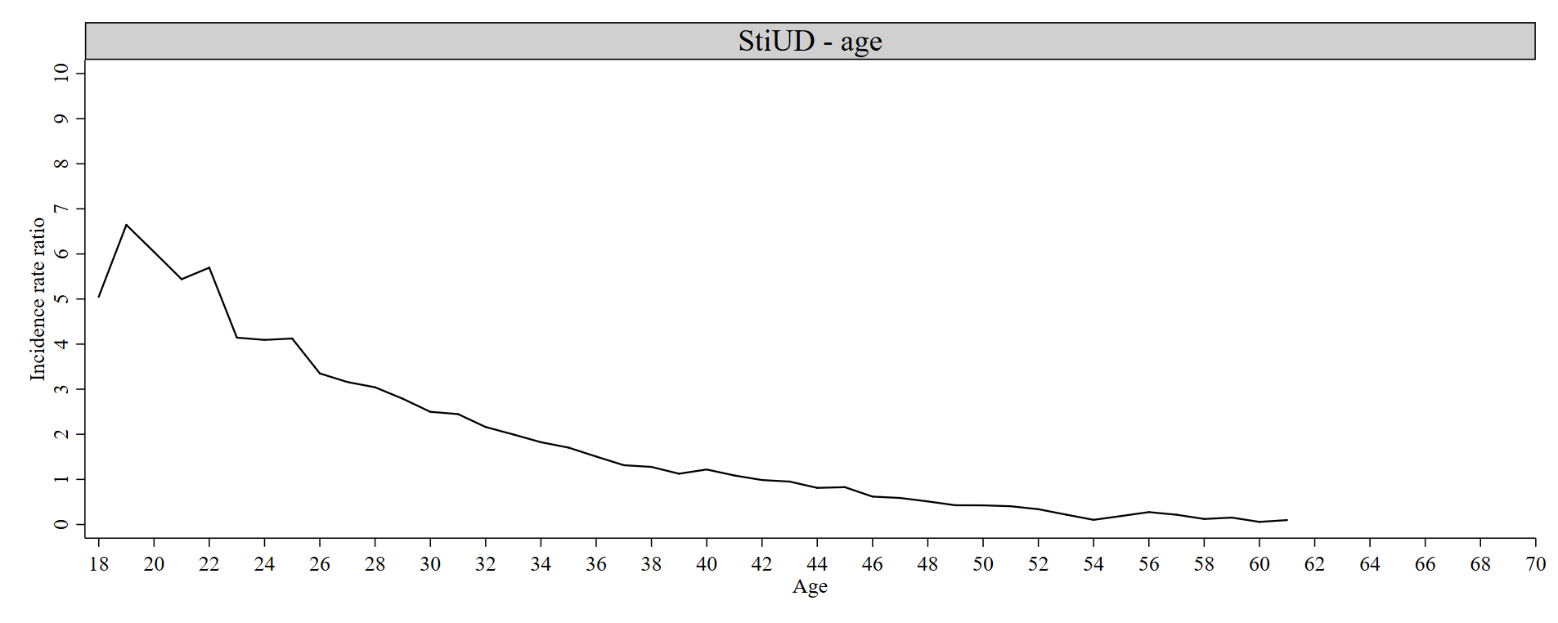


Supplementary Figure 9. Age effects on StiUD-related utilization rate, 1-year age groups (*IRR*)


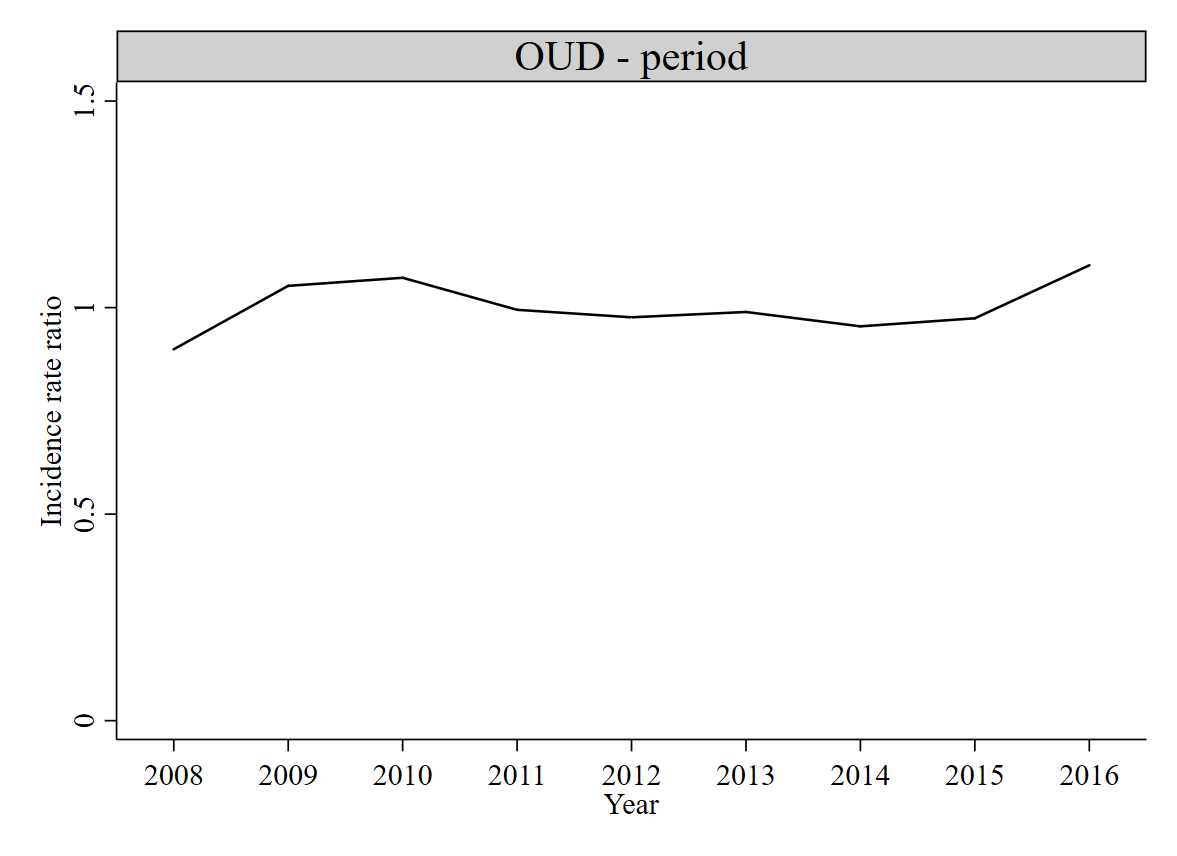


Supplementary Figure 10. Period effects on OUD-related utilization rate, 1-year period groups (*IRR*)


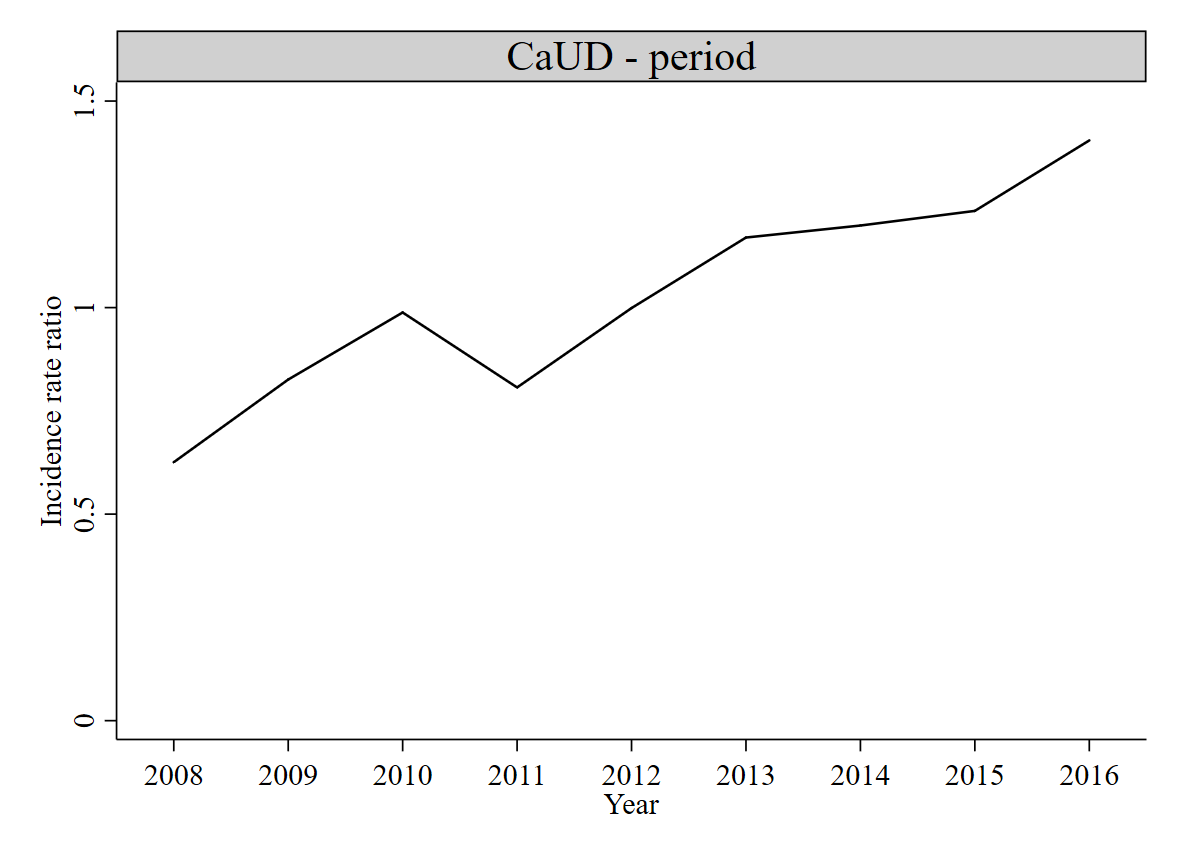


Supplementary Figure 11. Period effects on CaUD-related utilization rate, 1-year period groups (*IRR*)


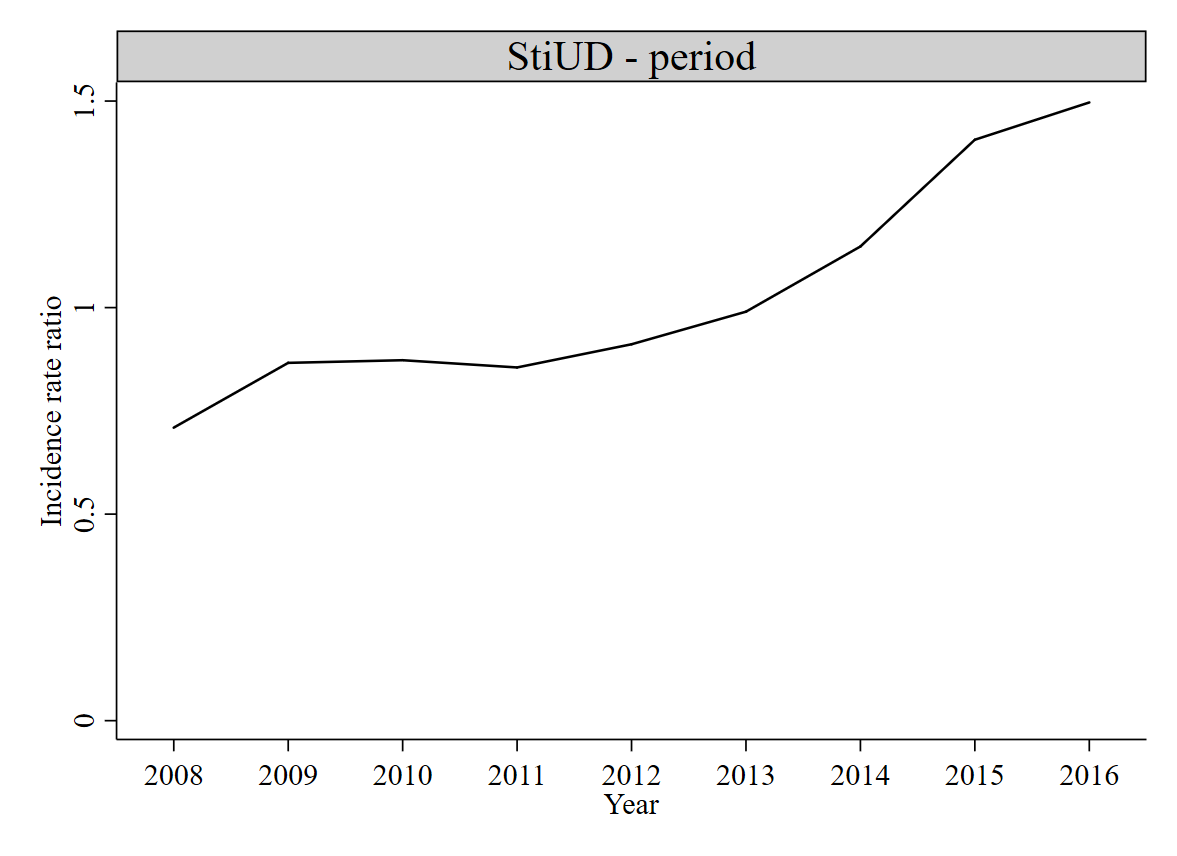


Supplementary Figure 12. Period effects on StiUD-related utilization rate, 1-year period groups (*IRR*)


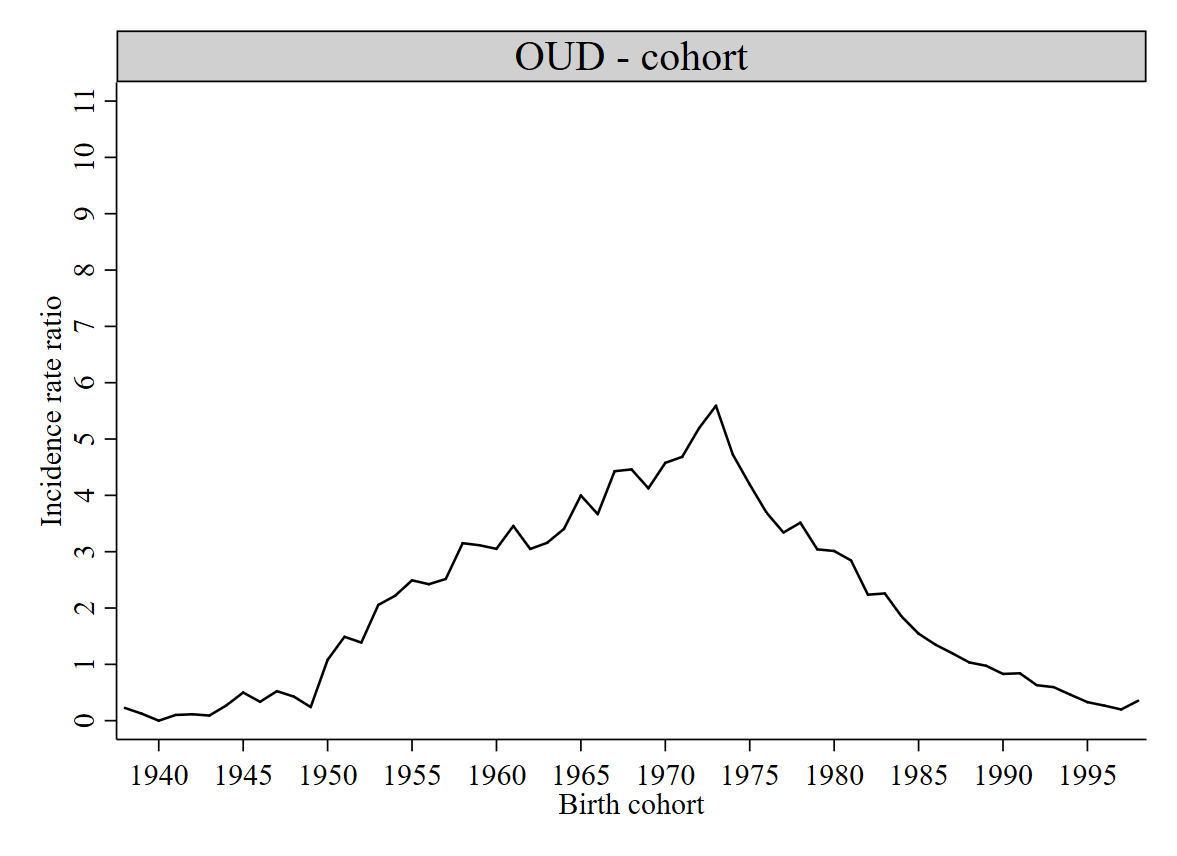


Supplementary Figure 13. Cohort effects on OUD-related utilization rate, 1-year cohort groups (*IRR*)


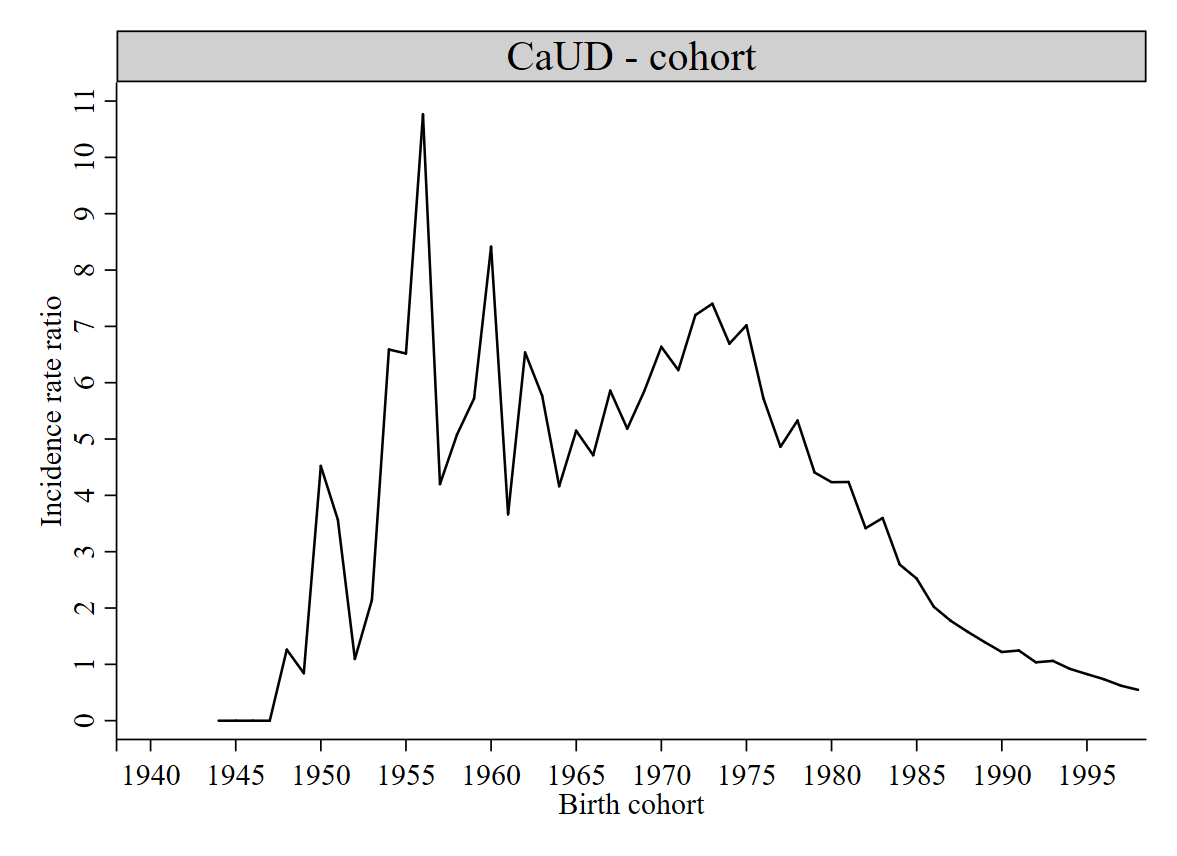


Supplementary Figure 14. Cohort effects on CaUD-related utilization rate, 1-year cohort groups (*IRR*)


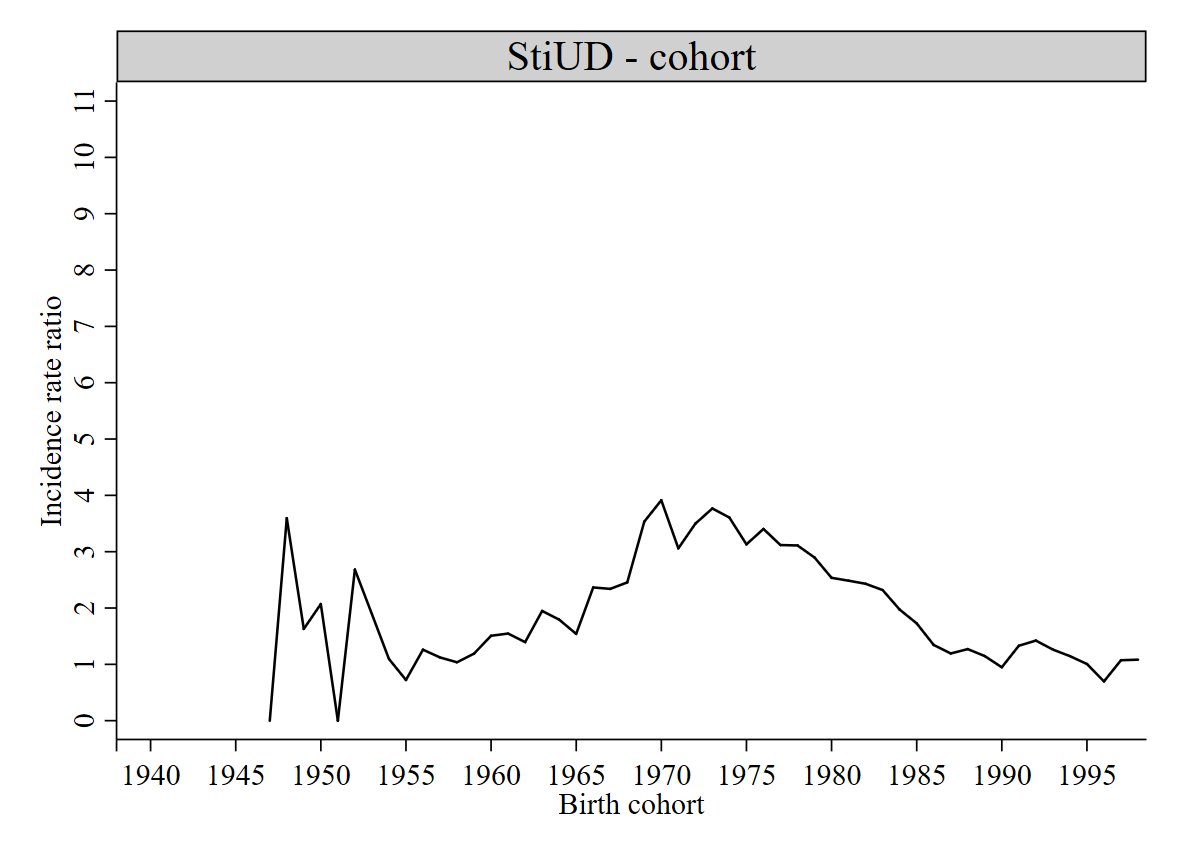


Supplementary Figure 15. Cohort effects on StiUD-related utilization rate, 1-year cohort groups (*IRR*)

Supplementary Table 2. Negative binomial APC models on OUD-, CaUD-, and StiUD-related utilization rate

|  |  | OUD | |  | CaUD | |  | StiUD | |
| --- | --- | --- | --- | --- | --- | --- | --- | --- | --- |
|  |  | *IRR* (95% *CI*) | *P* |  | *IRR* (95% *CI*) | *P* |  | *IRR* (95% *CI*) | *P* |
| Intercept |  | 0.00 (0.00, 0.76) | 0.043 |  | 0.00 (0.00, 14.30 * 10^15^) | 0.681 |  | 0.00 (0.00, 1.45 * 10^12^) | 0.593 |
| Age |  |  |  |  |  |  |  |  |  |
| 18 |  | 5.55 (0.00, 4,211,709.00) | 0.804 |  | 87.30 (0.00, 7.57 * 10^36^) | 0.913 |  | 5.05 (0.00, 0.26 * 10^27^) | 0.957 |
| 19 |  | 6.86 (0.00, 3,093,513.00) | 0.772 |  | 72.27 (0.00, 0.19 * 10^36^) | 0.913 |  | 6.65 (0.00, 22.00 * 10^24^) | 0.948 |
| 20 |  | 7.22 (0.00, 1,935,120.00) | 0.756 |  | 54.68 (0.00, 4.34 * 10^33^) | 0.915 |  | 6.05 (0.00, 1.27 * 10^24^) | 0.948 |
| 21 |  | 6.37 (0.00, 1,013,615.00) | 0.762 |  | 40.91 (0.00, 98.30 * 10^30^) | 0.917 |  | 5.44 (0.00, 73.00 *10^21^) | 0.948 |
| 22 |  | 5.31 (0.00, 501,665.60) | 0.775 |  | 27.72 (0.00, 2.02 * 10^30^) | 0.922 |  | 5.70 (0.00, 4.87 * 10^21^) | 0.944 |
| 23 |  | 4.51 (0.00, 253,067.20) | 0.787 |  | 21.15 (0.00, 46.60 * 10^27^) | 0.924 |  | 4.14 (0.00, 0.23 * 10^21^) | 0.951 |
| 24 |  | 3.77 (0.00, 125,945.20) | 0.803 |  | 15.28 (0.00, 1.02 * 10^27^) | 0.928 |  | 4.09 (0.00, 14.20 * 10^18^) | 0.948 |
| 25 |  | 3.49 (0.00, 69,194.81) | 0.804 |  | 11.50 (0.00, 23.20 * 10^24^) | 0.932 |  | 4.12 (0.00, 0.91 * 10^18^) | 0.945 |
| 26 |  | 2.99 (0.00, 35,235.60) | 0.819 |  | 8.67 (0.00, 0.53 * 10^24^) | 0.936 |  | 3.35 (0.00, 47.00 * 10^15^) | 0.949 |
| 27 |  | 2.70 (0.00, 18,891.90) | 0.826 |  | 6.57 (0.00, 12.10 * 10^21^) | 0.940 |  | 3.16 (0.00, 2.82 * 10^15^) | 0.948 |
| 28 |  | 2.40 (0.00, 9,977.13) | 0.837 |  | 5.66 (0.00, 0.32 * 10^21^) | 0.940 |  | 3.04 (0.00, 0.17 * 10^15^) | 0.945 |
| 29 |  | 2.08 (0.00, 5,133.86) | 0.854 |  | 4.64 (0.00, 7.86 * 10^18^) | 0.943 |  | 2.79 (0.00, 10.10 * 10^12^) | 0.945 |
| 30 |  | 1.93 (0.00, 2,836.57) | 0.860 |  | 3.88 (0.00, 0.20 * 10^18^) | 0.945 |  | 2.50 (0.00, 0.58 * 10^12^) | 0.945 |
| 31 |  | 1.73 (0.00, 1,513.20) | 0.873 |  | 3.35 (0.00, 5.19 * 10^15^) | 0.946 |  | 2.45 (0.00, 36.00 * 10^9^) | 0.940 |
| 32 |  | 1.77 (0.00, 919.39) | 0.857 |  | 2.89 (0.00, 0.14 *10^15^) | 0.947 |  | 2.16 (0.00, 2.02 * 10^9^) | 0.942 |
| 33 |  | 1.62 (0.01, 500.09) | 0.868 |  | 2.57 (0.00, 3.65 * 10^12^) | 0.947 |  | 2.00 (0.00, 0.12 * 10^9^) | 0.940 |
| 34 |  | 1.49 (0.01, 272.97) | 0.881 |  | 1.97 (0.00, 84.60 * 10^9^) | 0.957 |  | 1.82 (0.00, 6,924,877.00) | 0.938 |
| 35 |  | 1.43 (0.01, 155.25) | 0.882 |  | 1.90 (0.00, 2.48 * 10^9^) | 0.952 |  | 1.71 (0.00, 412,221.10) | 0.933 |
| 36 |  | 1.39 (0.02, 89.71) | 0.878 |  | 1.54 (0.00, 60.70 * 10^6^) | 0.961 |  | 1.51 (0.00, 23,203.02) | 0.934 |
| 37 |  | 1.33 (0.03, 51.13) | 0.879 |  | 1.47 (0.00, 1,760,661.00) | 0.957 |  | 1.32 (0.00, 1,291.76) | 0.938 |
| 38 |  | 1.27 (0.06, 28.97) | 0.882 |  | 1.15 (0.00, 41,472.25) | 0.980 |  | 1.28 (0.02, 80.07) | 0.908 |
| 39 |  | 1.13 (0.08, 15.37) | 0.927 |  | 0.92 (0.00, 1,003.95) | 0.980 |  | 1.13 (0.28, 4.56) | 0.867 |
| 40 |  | 1.14 (0.14, 9.21) | 0.904 |  | 1.01 (0.03, 33.48) | 0.998 |  | 1.22 (0.30, 4.95) | 0.780 |
| 41 |  | 1.02 (0.21, 4.94) | 0.977 |  | 0.65 (0.51, 0.82) | <0.001 |  | 1.09 (0.02, 68.23) | 0.968 |
| 42 |  | 0.97 (0.33, 2.79) | 0.948 |  | 0.71 (0.02, 23.63) | 0.848 |  | 0.99 (0.00, 969.27) | 0.997 |
| 43 |  | 0.90 (0.52, 1.57) | 0.715 |  | 0.54 (0.00, 597.07) | 0.865 |  | 0.95 (0.00, 14,673.01) | 0.992 |
| 44 |  | 0.86 (0.70, 1.05) | 0.138 |  | 0.55 (0.00, 19,786.19) | 0.910 |  | 0.81 (0.00, 196,711.30) | 0.974 |
| 45 |  | 0.81 (0.47, 1.42) | 0.467 |  | 0.49 (0.00, 582,013.40) | 0.920 |  | 0.83 (0.00, 3,145,206.00) | 0.981 |
| 46 |  | 0.78 (0.27, 2.26) | 0.653 |  | 0.37 (0.00, 14.40 * 10^6^) | 0.910 |  | 0.62 (0.00, 0.37 * 10^6^) | 0.958 |
| 47 |  | 0.73 (0.15, 3.54) | 0.699 |  | 0.36 (0.00, 0.47 * 10^9^) | 0.924 |  | 0.59 (0.00, 0.55 * 10^9^) | 0.960 |
| 48 |  | 0.69 (0.08, 5.56) | 0.724 |  | 0.37 (0.00, 16.10 * 10^9^) | 0.937 |  | 0.51 (0.00, 7.53 * 10^9^) | 0.955 |
| 49 |  | 0.70 (0.05, 9.56) | 0.791 |  | 0.24 (0.00, 0.34 * 10^12^) | 0.920 |  | 0.43 (0.00, 98.60 * 10^9^) | 0.949 |
| 50 |  | 0.64 (0.03, 14.62) | 0.779 |  | 0.22 (0.00, 10.30 * 10^12^) | 0.925 |  | 0.42 (0.00, 1.54 * 10^12^) | 0.954 |
| 51 |  | 0.66 (0.02, 25.26) | 0.821 |  | 0.22 (0.00, 0.34 * 10^15^) | 0.932 |  | 0.41 (0.00, 23.10 * 10^12^) | 0.955 |
| 52 |  | 0.53 (0.01, 34.12) | 0.763 |  | 0.17 (0.00, 8.60 * 10^15^) | 0.928 |  | 0.34 (0.00, 0.30 * 10^15^) | 0.951 |
| 53 |  | 0.61 (0.01, 66.14) | 0.835 |  | 0.11 (0.00, 0.18 * 10^18^) | 0.917 |  | 0.22 (0.00, 3.09 * 10^15^) | 0.936 |
| 54 |  | 0.53 (0.00, 97.62) | 0.813 |  | 0.12 (0.00, 6.55 * 10^18^) | 0.926 |  | 0.10 (0.00, 22.80 * 10^15^) | 0.911 |
| 55 |  | 0.52 (0.00, 161.07) | 0.824 |  | 0.12 (0.00, 0.22 * 10^21^) | 0.932 |  | 0.19 (0.00, 0.66 * 10^18^) | 0.939 |
| 56 |  | 0.51 (0.00, 264.14) | 0.832 |  | 0.10 (0.00, 6.30 * 10^21^) | 0.932 |  | 0.27 (0.00, 15.00 * 10^18^) | 0.956 |
| 57 |  | 0.44 (0.00, 382.25) | 0.811 |  | 0.09 (0.00, 0.17 * 10^24^) | 0.931 |  | 0.22 (0.00, 0.19 * 10^21^) | 0.950 |
| 58 |  | 0.49 (0.00, 720.57) | 0.848 |  | 0.03 (0.00, 2.19 * 10^24^) | 0.910 |  | 0.12 (0.00, 1.65 * 10^21^) | 0.936 |
| 59 |  | 0.38 (0.00, 952.30) | 0.811 |  | 0.10 (0.00, 0.23 * 10^27^) | 0.944 |  | 0.15 (0.00, 32.20 * 10^21^) | 0.945 |
| 60 |  | 0.44 (0.00, 1,848.45) | 0.849 |  | 0.04 (0.00, 2.66 * 10^27^) | 0.922 |  | 0.06 (0.00, 0.19 * 10^24^) | 0.921 |
| 61 |  | 0.34 (0.00, 2,350.99) | 0.809 |  | 0.04 (0.00, 22.90 * 10^27^) | 0.927 |  | 0.10 (0.00, 5.12 * 10^24^) | 0.939 |
| 62 |  | 0.42 (0.00, 4,944.89) | 0.856 |  | 0.09 (0.00, 6.90 * 10^30^) | 0.948 |  |  |  |
| 63 |  | 0.37 (0.00, 7,418.06) | 0.845 |  | 0.11 (0.00, 0.28 * 10^33^) | 0.954 |  |  |  |
| 64 |  | 0.25 (0.00, 8,451.29) | 0.796 |  | 0.14 (0.00, 12.40 * 10^33^) | 0.962 |  |  |  |
| 65 |  | 0.33 (0.00, 18,566.49) | 0.842 |  |  |  |  |  |  |
| 66 |  | 0.21 (0.00, 20,285.09) | 0.792 |  |  |  |  |  |  |
| 67 |  | 0.39 (0.00, 62,775.76) | 0.879 |  |  |  |  |  |  |
| 68 |  | 0.23 (0.00, 63,233.06) | 0.820 |  |  |  |  |  |  |
| 69 |  | 0.17 (0.00, 76,144.38) | 0.788 |  |  |  |  |  |  |
| 70 |  | 0.42 (0.00, 323,837.90) | 0.901 |  |  |  |  |  |  |
| Period |  |  |  |  |  |  |  |  |  |
| 2008 |  | 0.90 (0.11, 7.23) | 0.920 |  | 0.63 (0.00, 745,827.00) | 0.948 |  | 0.71 (0.00, 43,197.64) | 0.951 |
| 2009 |  | 1.05 (0.22, 5.03) | 0.949 |  | 0.83 (0.00, 29,795.10) | 0.971 |  | 0.87 (0.00, 3,358.46) | 0.973 |
| 2010 |  | 1.07 (0.38, 3.04) | 0.896 |  | 0.99 (0.00, 1,079.30) | 0.997 |  | 0.87 (0.00, 215.47) | 0.961 |
| 2011 |  | 0.99 (0.59, 1.68) | 0.984 |  | 0.81 (0.02, 26.69) | 0.904 |  | 0.86 (0.05, 13.46) | 0.911 |
| 2012 |  | 0.98 (0.91, 1.05) | 0.499 |  | 1.00 (0.91, 1.09) | 0.979 |  | 0.91 (0.83, 1.01) | 0.066 |
| 2013 |  | 0.99 (0.59, 1.67) | 0.968 |  | 1.17 (0.04, 38.69) | 0.930 |  | 0.99 (0.06, 15.58) | 0.994 |
| 2014 |  | 0.95 (0.34, 2.71) | 0.931 |  | 1.20 (0.00, 1,309.28) | 0.959 |  | 1.15 (0.00, 283.45) | 0.961 |
| 2015 |  | 0.97 (0.20, 4.65) | 0.974 |  | 1.23 (0.00, 44,527.05) | 0.969 |  | 1.41 (0.00, 5,452.15) | 0.936 |
| 2016 |  | 1.10 (0.14, 8.86) | 0.927 |  | 1.40 (0.00, 1,674,339.00) | 0.962 |  | 1.50 (0.00, 91,121.93) | 0.943 |
| Cohort |  |  |  |  |  |  |  |  |  |
| 1938 |  | 0.23 (0.00, 14.50 * 10^9^) | 0.907 |  |  |  |  |  |  |
| 1939 |  | 0.12 (0.00, 4.72 * 10^9^) | 0.867 |  |  |  |  |  |  |
| 1940 |  | 0.00 (0.00, 0.26 * 10^228^) | 0.953 |  |  |  |  |  |  |
| 1941 |  | 0.10 (0.00, 1.35 * 10^9^) | 0.847 |  |  |  |  |  |  |
| 1942 |  | 0.11 (0.00, 0.90 * 10^9^) | 0.852 |  |  |  |  |  |  |
| 1943 |  | 0.09 (0.00, 0.43 * 10^9^) | 0.833 |  |  |  |  |  |  |
| 1944 |  | 0.27 (0.00, 0.75 * 10^9^) | 0.906 |  | 0.00 (0.00, > 10^308^) | 0.988 |  |  |  |
| 1945 |  | 0.50 (0.00, 0.82 * 10^9^) | 0.949 |  | 0.00 (0.00, > 10^308^) | 0.984 |  |  |  |
| 1946 |  | 0.33 (0.00, 0.33 * 10^9^) | 0.917 |  | 0.00 (0.00, > 10^308^) | 0.980 |  |  |  |
| 1947 |  | 0.52 (0.00, 0.30 * 10^9^) | 0.950 |  | 0.00 (0.00, > 10^308^) | 0.977 |  | 0.00 (0.00, > 10^308^) | 0.987 |
| 1948 |  | 0.43 (0.00, 0.15 * 10^9^) | 0.933 |  | 1.26 (0.00, 9.95 * 10^57^) | 0.997 |  | 3.59 (0.00, 36.40 * 10^45^) | 0.981 |
| 1949 |  | 0.24 (0.00, 49.50 * 10^6^) | 0.884 |  | 0.84 (0.00, 0.20 * 10^57^) | 0.998 |  | 1.63 (0.00, 1.05 * 10^45^) | 0.993 |
| 1950 |  | 1.08 (0.00, 0.13 * 10^9^) | 0.994 |  | 4.52 (0.00, 32.60 * 10^54^) | 0.981 |  | 2.07 (0.00, 84.90 * 10^42^) | 0.989 |
| 1951 |  | 1.49 (0.00, 0.11 * 10^9^) | 0.966 |  | 3.57 (0.00, 0.78 * 10^54^) | 0.984 |  | 0.00 (0.00, > 10^308^) | 0.970 |
| 1952 |  | 1.39 (0.00, 59.40 * 10^6^) | 0.971 |  | 1.10 (0.00, 7.26 * 10^51^) | 0.999 |  | 2.68 (0.00, 0.45 * 10^42^) | 0.984 |
| 1953 |  | 2.05 (0.00, 52.30 * 10^6^) | 0.934 |  | 2.14 (0.00, 0.43 * 10^51^) | 0.990 |  | 1.89 (0.00, 20.00 * 10^39^) | 0.989 |
| 1954 |  | 2.22 (0.00, 33.60 * 10^6^) | 0.925 |  | 6.59 (0.00, 40.00 * 10^48^) | 0.974 |  | 1.10 (0.00, 0.74 * 10^39^) | 0.998 |
| 1955 |  | 2.49 (0.00, 22.40 * 10^6^) | 0.911 |  | 6.52 (0.00, 1.20 * 10^48^) | 0.973 |  | 0.72 (0.00, 31.10 * 10^36^) | 0.994 |
| 1956 |  | 2.42 (0.00, 12.90 * 10^6^) | 0.911 |  | 10.76 (0.00, 59.90 * 10^45^) | 0.965 |  | 1.26 (0.00, 3.46 * 10^36^) | 0.996 |
| 1957 |  | 2.52 (0.00, 7,981,903.00) | 0.904 |  | 4.20 (0.00, 0.71 * 10^45^) | 0.978 |  | 1.12 (0.00, 0.20 * 10^36^) | 0.998 |
| 1958 |  | 3.15 (0.00, 5,939,561.00) | 0.876 |  | 5.08 (0.00, 25.90 * 10^42^) | 0.974 |  | 1.04 (0.00, 11.60 * 10^33^) | 0.999 |
| 1959 |  | 3.11 (0.00, 3,487,570.00) | 0.873 |  | 5.72 (0.00, 0.89 * 10^42^) | 0.971 |  | 1.19 (0.00, 0.85 * 10^33^) | 0.996 |
| 1960 |  | 3.05 (0.00, 2,029,828.00) | 0.870 |  | 8.41 (0.00, 39.40 * 10^39^) | 0.964 |  | 1.51 (0.00, 68.20 * 10^30^) | 0.991 |
| 1961 |  | 3.46 (0.00, 1,366,544.00) | 0.850 |  | 3.66 (0.00, 0.52 * 10^39^) | 0.977 |  | 1.55 (0.00, 4.46 * 10^30^) | 0.990 |
| 1962 |  | 3.05 (0.00, 715,900.10) | 0.860 |  | 6.54 (0.00, 28.10 * 10^36^) | 0.965 |  | 1.39 (0.00, 0.26 * 10^30^) | 0.992 |
| 1963 |  | 3.16 (0.00, 440,584.60) | 0.849 |  | 5.77 (0.00, 0.75 * 10^36^) | 0.966 |  | 1.95 (0.00, 22.90 * 10^27^) | 0.984 |
| 1964 |  | 3.41 (0.00, 282,353.20) | 0.832 |  | 4.16 (0.00, 16.40 * 10^33^) | 0.971 |  | 1.79 (0.00, 1.34 * 10^27^) | 0.985 |
| 1965 |  | 4.00 (0.00, 196,940.90) | 0.802 |  | 5.15 (0.00, 0.62 * 10^33^) | 0.965 |  | 1.54 (0.00, 73.60 * 10^24^) | 0.989 |
| 1966 |  | 3.67 (0.00, 107,348.70) | 0.804 |  | 4.71 (0.00, 17.10 * 10^30^) | 0.966 |  | 2.37 (0.00, 7.21 * 10^24^) | 0.976 |
| 1967 |  | 4.43 (0.00, 77,031.82) | 0.765 |  | 5.86 (0.00, 0.65 * 10^30^) | 0.959 |  | 2.34 (0.00, 0.46 * 10^24^) | 0.975 |
| 1968 |  | 4.46 (0.00, 46,097.48) | 0.751 |  | 5.18 (0.00, 17.30 * 10^27^) | 0.959 |  | 2.45 (0.00, 30.50 * 10^21^) | 0.972 |
| 1969 |  | 4.13 (0.00, 25,334.90) | 0.750 |  | 5.85 (0.00, 0.59 * 10^27^) | 0.954 |  | 3.53 (0.00, 2.80 * 10^21^) | 0.959 |
| 1970 |  | 4.58 (0.00, 16,699.50) | 0.716 |  | 6.64 (0.00, 20.40 * 10^24^) | 0.948 |  | 3.91 (0.00, 0.20 * 10^21^) | 0.953 |
| 1971 |  | 4.68 (0.00, 10,154.97) | 0.694 |  | 6.22 (0.00, 0.58 * 10^24^) | 0.946 |  | 3.06 (0.00, 9.91 * 10^18^) | 0.959 |
| 1972 |  | 5.19 (0.00, 6,689.15) | 0.652 |  | 7.20 (0.00, 20.40 * 10^21^) | 0.938 |  | 3.50 (0.00, 0.72 * 10^18^) | 0.951 |
| 1973 |  | 5.59 (0.01, 4,278.88) | 0.611 |  | 7.40 (0.00, 0.64 * 10^21^) | 0.932 |  | 3.77 (0.00, 49.90 * 10^15^) | 0.944 |
| 1974 |  | 4.73 (0.01, 2,149.18) | 0.619 |  | 6.69 (0.00, 17.50 * 10^18^) | 0.930 |  | 3.61 (0.00, 3.06 * 10^15^) | 0.942 |
| 1975 |  | 4.19 (0.02, 1,133.21) | 0.616 |  | 7.02 (0.00, 0.56 * 10^18^) | 0.922 |  | 3.13 (0.00, 0.17 * 10^15^) | 0.944 |
| 1976 |  | 3.69 (0.02, 592.93) | 0.614 |  | 5.72 (0.00, 13.80 * 10^15^) | 0.923 |  | 3.40 (0.00, 11.90 * 10^12^) | 0.934 |
| 1977 |  | 3.34 (0.03, 318.88) | 0.604 |  | 4.86 (0.00, 0.36 * 10^15^) | 0.923 |  | 3.12 (0.00, 0.70 * 10^12^) | 0.932 |
| 1978 |  | 3.52 (0.06, 199.44) | 0.542 |  | 5.33 (0.00, 12.00 * 10^12^) | 0.908 |  | 3.11 (0.00, 45.00 * 10^9^) | 0.924 |
| 1979 |  | 3.04 (0.09, 102.59) | 0.535 |  | 4.41 (0.00, 0.30 * 10^12^) | 0.907 |  | 2.90 (0.00, 2.71 * 10^9^) | 0.920 |
| 1980 |  | 3.01 (0.15, 60.40) | 0.471 |  | 4.23 (0.00, 8.99 * 10^9^) | 0.895 |  | 2.54 (0.00, 0.15 * 10^9^) | 0.919 |
| 1981 |  | 2.84 (0.24, 33.92) | 0.408 |  | 4.24 (0.00, 0.28 * 10^9^) | 0.875 |  | 2.49 (0.00, 9,898,105.00) | 0.907 |
| 1982 |  | 2.24 (0.32, 15.87) | 0.421 |  | 3.42 (0.00, 7,011,048.00) | 0.868 |  | 2.43 (0.00, 641,433.00) | 0.889 |
| 1983 |  | 2.26 (0.53, 9.55) | 0.268 |  | 3.60 (0.00, 235,336.20) | 0.821 |  | 2.32 (0.00, 41,462.38) | 0.866 |
| 1984 |  | 1.85 (0.73, 4.67) | 0.194 |  | 2.77 (0.00, 6,065.59) | 0.795 |  | 1.97 (0.00, 2,505.16) | 0.852 |
| 1985 |  | 1.55 (1.01, 2.37) | 0.046 |  | 2.52 (0.03, 215.74) | 0.684 |  | 1.73 (0.02, 177.62) | 0.817 |
| 1986 |  | 1.35 (1.08, 1.68) | 0.007 |  | 2.02 (0.22, 18.57) | 0.533 |  | 1.34 (0.10, 18.99) | 0.826 |
| 1987 |  | 1.20 (0.61, 2.35) | 0.604 |  | 1.77 (0.04, 79.91) | 0.768 |  | 1.19 (0.07, 19.24) | 0.901 |
| 1988 |  | 1.03 (0.32, 3.39) | 0.955 |  | 1.58 (0.00, 1,675.04) | 0.898 |  | 1.27 (0.01, 163.97) | 0.923 |
| 1989 |  | 0.97 (0.18, 5.35) | 0.977 |  | 1.39 (0.00, 43,490.76) | 0.950 |  | 1.15 (0.00, 1866.33) | 0.971 |
| 1990 |  | 0.83 (0.09, 7.66) | 0.870 |  | 1.22 (0.00, 1,184,960.00) | 0.977 |  | 0.95 (0.00, 21,851.98) | 0.992 |
| 1991 |  | 0.84 (0.05, 13.06) | 0.902 |  | 1.25 (0.00, 38.60 * 10^6^) | 0.980 |  | 1.33 (0.00, 453,796.80) | 0.965 |
| 1992 |  | 0.63 (0.02, 16.45) | 0.781 |  | 1.04 (0.00, 1.04 * 10^9^) | 0.997 |  | 1.42 (0.00, 7,337,276.00) | 0.964 |
| 1993 |  | 0.60 (0.01, 26.17) | 0.788 |  | 1.06 (0.00, 34.50 * 10^9^) | 0.996 |  | 1.26 (0.00, 99.60 * 10^6^) | 0.980 |
| 1994 |  | 0.46 (0.01, 34.14) | 0.725 |  | 0.92 (0.00, 0.98 * 10^12^) | 0.995 |  | 1.15 (0.00, 1.39 * 10^9^) | 0.990 |
| 1995 |  | 0.33 (0.00, 41.07) | 0.652 |  | 0.83 (0.00, 28.70 * 10^12^) | 0.990 |  | 1.01 (0.00, 18.90 * 10^9^) | 1.000 |
| 1996 |  | 0.27 (0.00, 56.39) | 0.630 |  | 0.74 (0.00, 0.84 * 10^15^) | 0.986 |  | 0.70 (0.00, 0.20 * 10^12^) | 0.979 |
| 1997 |  | 0.20 (0.00, 70.93) | 0.591 |  | 0.62 (0.00, 23.20 * 10^15^) | 0.980 |  | 1.07 (0.00, 4.88 * 10^12^) | 0.996 |
| 1998 |  | 0.35 (0.00, 213.66) | 0.750 |  | 0.55 (0.00, 0.68 * 10^18^) | 0.977 |  | 1.08 (0.00, 76.90 * 10^12^) | 0.996 |
| Observations |  | 477 |  |  | 423 |  |  | 396 |  |
| BIC |  | –2,181.61 |  |  | –1,883.34 |  |  | –1,734.30 |  |

*Note*. APC = age, period, and cohort; OUD = opioids use disorders; CaUD = cannabis use disorders, StiUD = stimulants/cocaine use disorders; *IRR* = incidence rate ratio; *CI* = confidence interval; BIC = Bayesian information criterion.

Supplementary Table 3. Effect estimates that would not hold up at an alpha level of 0.01

|  | Age groups | Period groups | Cohort groups |
| --- | --- | --- | --- |
| AUD-related utilization | 20, 25, 26, 28, 36, 68 | 2009, 2012, 2013 | 1938, 1962, 1964, 1966 |
| ISUD-related utilization | 38, 46 | - | 1954, 1989, 1991 |
| OUD-/CaUD-,/StiUD-related utilization | - | - | - |

*Note*. AUD = alcohol use disorders; ISUD = illicit substances use disorders; OUD = opioids use disorders; CaUD = cannabis use disorders; StiUD = stimulants/cocaine use disorders.
